# Early diagnosis of breast cancer lung metastasis by nanoprobe-based luminescence imaging of the pre-metastatic niche

**DOI:** 10.1186/s12951-022-01346-4

**Published:** 2022-03-15

**Authors:** Hanwen Zheng, Chunsen Yuan, Jiajun Cai, Wendan Pu, Peng Wu, Chenwen Li, Gang Li, Yang Zhang, Jianxiang Zhang, Jiawei Guo, Dingde Huang

**Affiliations:** 1 Department of Nuclear Medicine, Southwest Hospital, Third Military Medical University (Army Medical University), 30 Gaotanyan Main Street, Chongqing, 400038 China; 2 Department of Pharmaceutics, College of Pharmacy, Third Military Medical University (Army Medical University), 30 Gaotanyan Main Street, Chongqing, 400038 China; 3 Department of Pharmaceutical Analysis, College of Pharmacy, Third Military Medical University (Army Medical University), 30 Gaotanyan Main Street, Chongqing, 400038 China; 4 College of Pharmacy and Medical Technology, Hanzhong Vocational and Technical College, Hanzhong, 723000 Shaanxi China; 5 State Key Laboratory of Trauma, Burn and Combined Injury, Third Military Medical University (Army Medical University), Chongqing, 400038 China

**Keywords:** Lung metastasis, Breast cancer, Luminescence imaging, Targeting nanoprobe, Neutrophils

## Abstract

**Background:**

Early detection of breast cancer lung metastasis remains highly challenging, due to few metastatic cancer cells at an early stage. Herein we propose a new strategy for early diagnosis of lung metastasis of breast cancer by luminescence imaging of pulmonary neutrophil infiltration via self-illuminating nanoprobes.

**Methods:**

Luminescent nanoparticles (LAD NPs) were engineered using a biocompatible, neutrophil-responsive self-illuminating cyclodextrin material and an aggregation-induced emission agent. The chemiluminescence resonance energy transfer (CRET) effect and luminescence properties of LAD NPs were fully characterized. Using mouse peritoneal neutrophils, in vitro luminescence properties of LAD NPs were thoroughly examined. In vivo luminescence imaging and correlation analyses were performed in mice inoculated with 4T1 cancer cells. Moreover, an active targeting nanoprobe was developed by surface decoration of LAD NPs with a neutrophil-targeting peptide, which was also systemically evaluated by in vitro and in vivo studies.

**Results:**

LAD NPs can generate long-wavelength and persistent luminescence due to the CRET effect. In a mouse model of 4T1 breast cancer lung metastasis, we found desirable correlation between neutrophils and tumor cells in the lungs, demonstrating the effectiveness of early imaging of the pre-metastatic niche by the newly developed LAD NPs. The active targeting nanoprobe showed further enhanced luminescence imaging capability for early detection of pulmonary metastasis. Notably, the targeting nanoprobe-based luminescence imaging strategy remarkably outperformed PET/CT imaging modalities in the examined mouse model. Also, preliminary tests demonstrated good safety of LAD NPs.

**Conclusions:**

The neutrophil-targeting imaging strategy based on newly developed luminescence nanoparticles can serve as a promising modality for early diagnosis of lung metastasis of breast cancers.

**Graphical Abstract:**

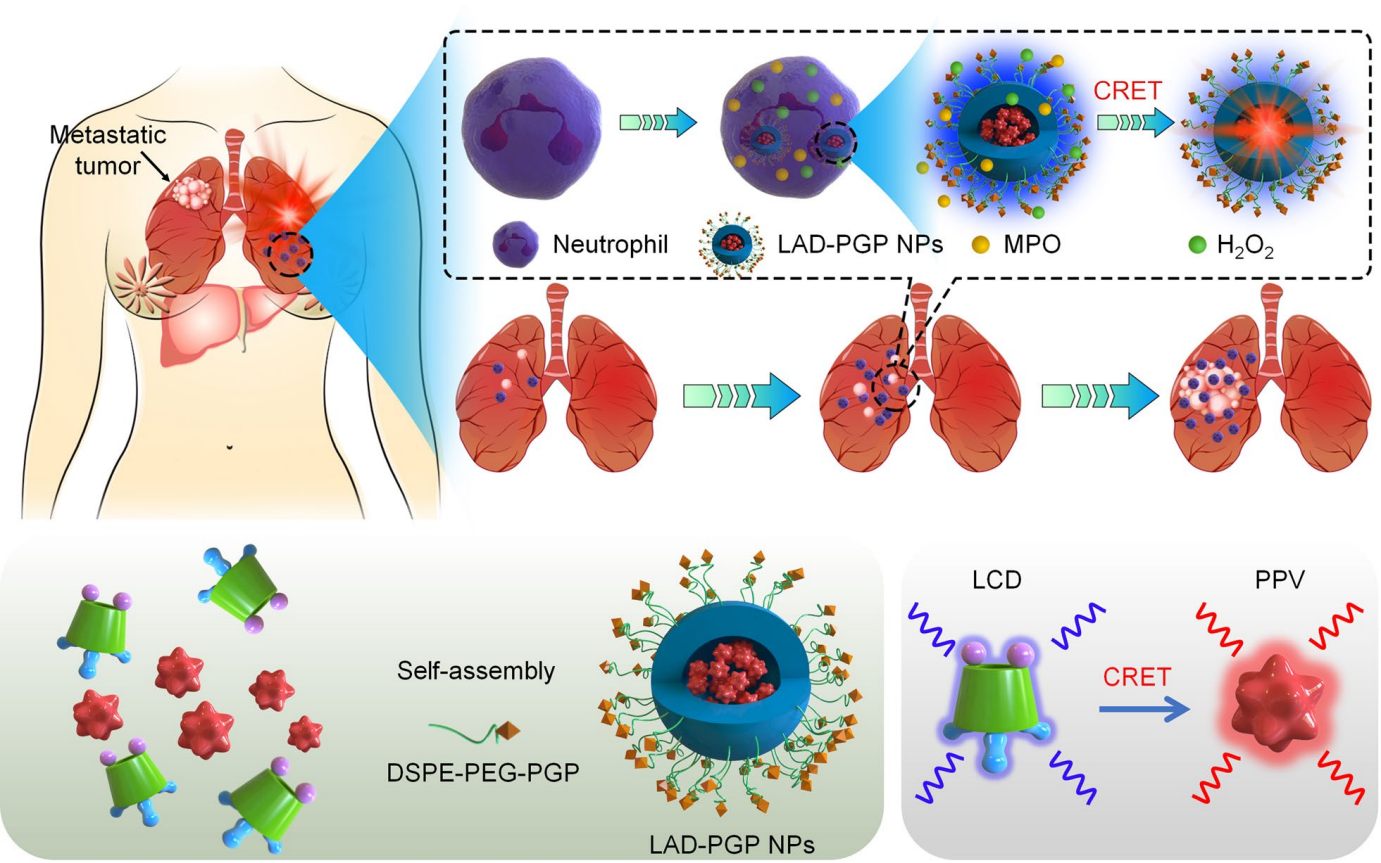

**Supplementary Information:**

The online version contains supplementary material available at 10.1186/s12951-022-01346-4.

## Background

Breast cancer remains the most frequently diagnosed cancer in women worldwide, and the majority of deaths caused by breast cancer are attributed to metastasis-related complications. Nearly 50% of breast cancer patients will develop metastasis [1–3]. The lung, bone, brain, and liver are preferential metastasis organs for breast cancer, termed the organ tropism. Extensive clinical studies in breast cancer patients with metastases have demonstrated that the lung is the primary organ of metastasis, which tends to occur within 5 years of initial breast cancer diagnosis [4, 5]. In addition, a high mortality rate of 60–70% for lung metastasis is of particular concern. Currently, effective therapeutic treatment of lung metastases remains highly challenging, largely resulting from the deficiency of early diagnostic methods. Accurate identification of the formation of lung micrometastases or pre-metastatic niches at an early stage of notable lung metastasis is of great significance for early diagnosis, symptomatic treatment, and prognosis of breast cancer lung metastasis.

Clinically, computed tomography (CT) and positron emission computed tomography (PET) are generally used for diagnosis of lung metastasis. However, CT cannot provide desirable resolution for micrometastases with size less than 2 mm. In addition, the radiotracer accumulation in micrometastases is far below the detectable threshold of PET/CT imaging [6–8]. Meanwhile, inevitable anxiety from cancer patients owing to the use of radioactive isotopes such as ^18^F-FDG cannot be ignored, despite relatively low radiation doses clinically used [9]. Whereas puncture biopsy of the lung tissue is still the gold standard for lung metastasis detection [10], it is time-consuming and invasive. In particular, puncture biopsy may lead to serious complications such as pneumothorax [11]. During the process of metastasis, less than 0.01% of primary tumor “seed” cells complete the metastatic cascade to develop macrometastases at the selected “soil” organs [12]. Consequently, it is extremely difficult to detect a few metastatic cancer cells at an early stage in the secondary organs by existing clinical imaging modalities. Other innovative strategies remain to be established.

It has been well documented that neutrophils play a critical role in the pre-metastatic niche formation and subsequent metastasis in different organs due to their pro-metastatic functions [13–16]. Neutrophil recruitment, induced by metastatic seeding of circulating tumor cells in the lungs, is closely associated with the formation of supportive metastatic inflammatory microenvironments [4, 13, 14, 17, 18]. Consequently, pulmonary infiltrated neutrophils might serve as an alternative target for detection of lung metastasis of breast cancer, while accurate, noninvasive, and real-time monitoring of lung neutrophils can provide a potentially promising strategy for early diagnosis of breast cancer lung metastasis. Most recent studies have demonstrated that luminescence imaging is promising for dynamically detecting neutrophils in different disease models varying from inflammation to tumors [19–22]. In this aspect, both small-molecular and nanoparticle probes have been developed and used for luminescence imaging of neutrophil-associated diseases [23–29]. Nevertheless, most self-luminescent agents exhibit short emission wavelengths, thereby leading to poor tissue penetration and undesirable deep-tissue imaging performance. To overcome this limitation, nanotechnology has been integrated with chemiluminescence resonance energy transfer (CRET) to afford effective self-illuminating nanoprobes with good deep-tissue imaging capacity [24, 30–32]. In particular, much attention has been paid to the development of high-performance luminescent nanoprobes for imaging diverse diseases, by the combination of self-illuminating molecules with aggregation-induced emission (AIE) fluorogens (i.e., AIEgens) [24, 33–39]. However, it remains unclear whether early diagnosis of lung metastasis can be realized by luminescence imaging via rationally designed luminescent nanoprobes.

**Fig. 1:**
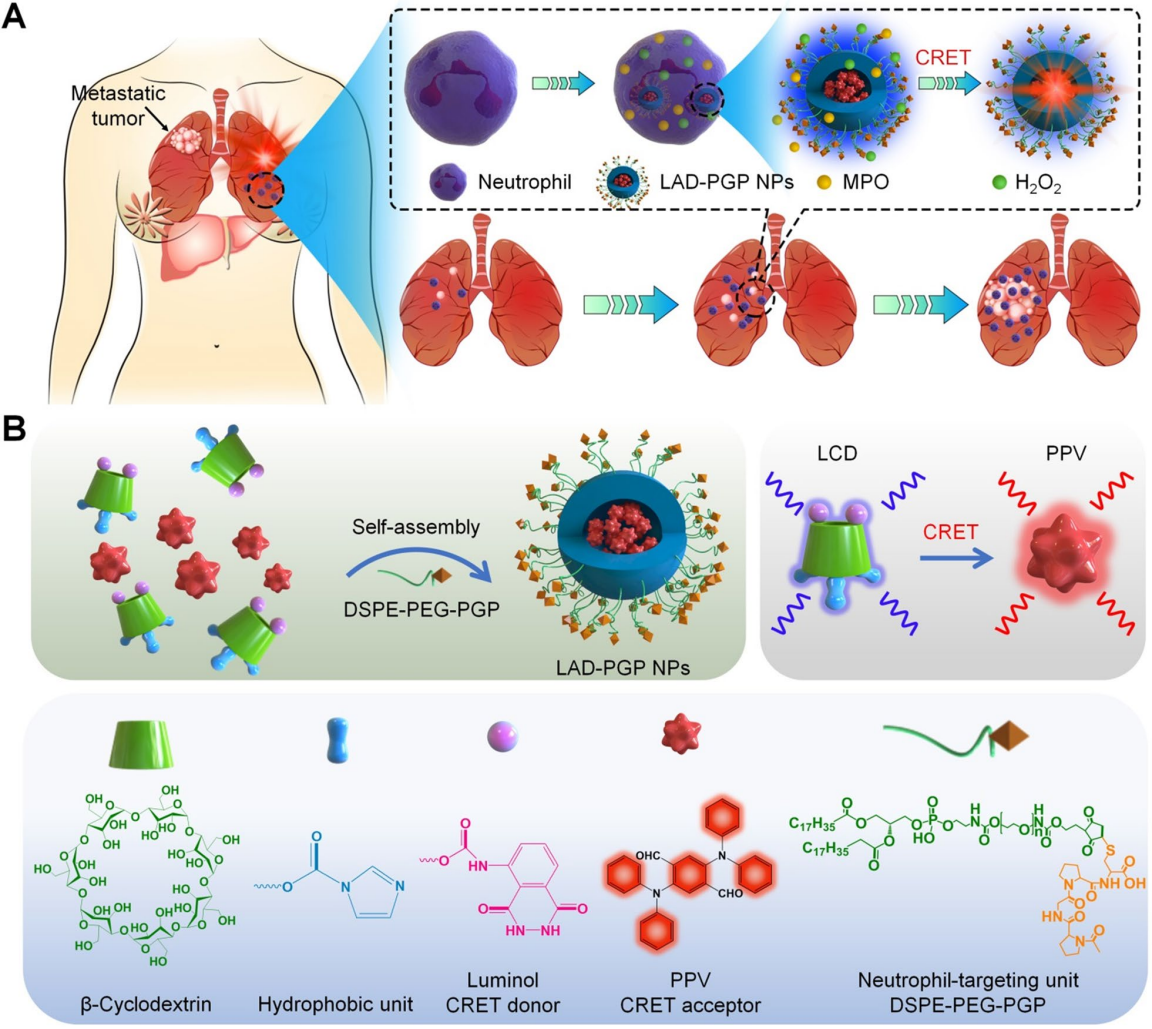
Schematic illustration of early imaging of pulmonary metastasis of breast cancer by a self-luminescence nanoprobe. **A** Neutrophil-dependent luminescence imaging of lung micrometastasis of breast cancer based on MPO/H_2_O_2_-mediated chemiluminescence resonance energy transfer (CRET) using a self-illuminating and neutrophil-targeting nanoprobe (LAD-PGP NPs). **B** A sketch shows engineering of LAD-PGP NPs composed of a self-luminescent material of luminol-conjugated β-cyclodextrin (LCD, a CRET donor), an aggregation-induced emission compound (PPV, a CRET acceptor), and a neutrophil-targeting unit DSPE-PEG-PGP

Herein we hypothesize that early diagnosis/detection of lung metastasis of breast cancer can be achieved by in vivo luminescence imaging of infiltrated neutrophils in the lung using self-illuminating nanoprobes with AIEgen-derived CRET capability. As a proof of concept, nanoparticles were first engineered using a biocompatible and neutrophil-triggerable luminescent material based on luminol-conjugated cyclodextrin, into which an AIE agent PPV was loaded to afford a self-luminescent CRET nanoprobe (Fig. 1). In a mouse model of breast cancer lung metastasis, we found the correlation between neutrophil and tumor cell counts, and demonstrated the feasibility of imaging early lung metastasis via the newly engineered nanoprobe. Moreover, luminescence imaging performance of the CRET nanoprobe can be further improved by surface engineering with a neutrophil-targeting peptide.

## Results and discussion

### Engineering of self-illuminating nanoparticles based on a luminescent material and an aggregation-induced emission (AIE) fluorogen

**Fig. 2:**
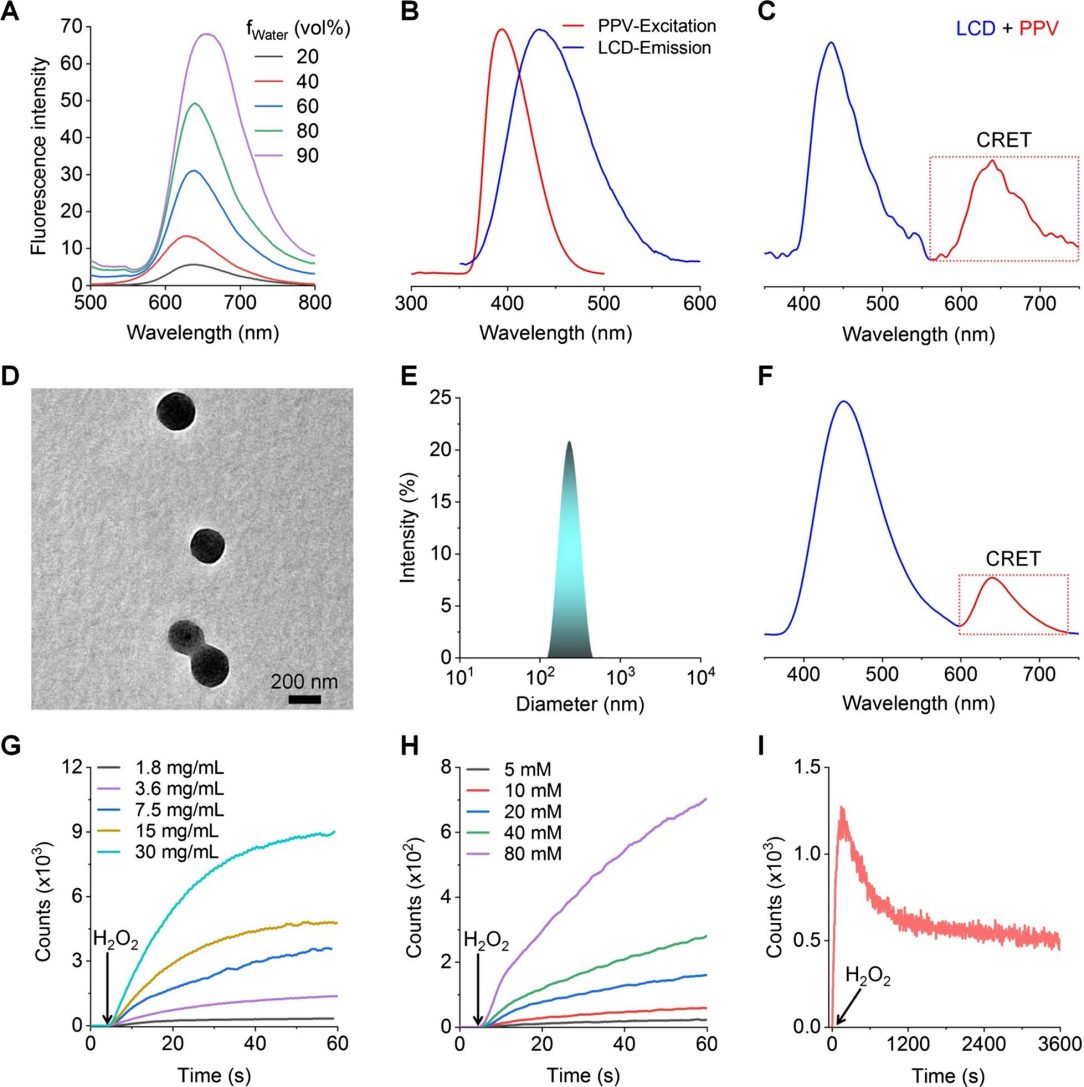
Characterization of luminescent properties of LCD and LCD/PPV NPs. **A** Fluorescence spectra of PPV in a solvent mixture of H_2_O/THF with different proportions of water (f_Water_) at an excitation wavelength (λ_ex_) of 425 nm. **B** The emission spectrum of LCD in the presence of hypochlorite (ClO^−^) and the excitation spectrum of PPV (λ_em_ = 650 nm). **C** The luminescence spectrum of LCD/PPV mixture in DMF/H_2_O at 100 mM ClO^−^. **D**, **E** TEM image (**D**) and size distribution (**E**) of LAD NPs. **F** The luminescence spectrum of LAD NPs at 100 mM ClO^−^. **G** Time-dependent luminescence signals of various concentrations of LAD NPs upon incubation with 10 mM H_2_O_2_. **H** Time-lapse luminescence curves of 10 mg/mL LAD NPs upon incubation with various levels of H_2_O_2_. **I** A typical time-resolved luminescence curve showing sustained luminescence of LAD NPs in presence of H_2_O_2_. The photon counts were acquired immediately after 10 mg/mL LAD NPs were incubated with 80 mM H_2_O_2_

A luminescent material (defined as LCD) was first synthesized by conjugating luminol onto β-cyclodextrin (β-CD) [26]. Of note, β-CD and its derivatives have been widely used in pharmaceutical preparations and the development of functional biomaterials [40]. LCD can be easily processed into well-defined nanoparticles with desirable luminescence properties for imaging acute inflammatory diseases. Measurements by Fourier-transform infrared (FT-IR), UV–visible, ^1^H NMR, and matrix-assisted laser desorption/ionization time-of-flight (MALDI–TOF) mass spectrometry revealed successful conjugation of luminol on β-CD (Additional file 1: Fig. S1). According to the ^1^H NMR spectrum, the product LCD exhibited characteristic proton peaks (3–6 ppm) due to –H and –OH moieties in β-CD. Proton signals at 6–9 ppm, which are characteristic peaks of aromatic rings in imidazole and luminol groups, were also clearly observed. Calculation based on the ^1^H NMR spectrum indicated that approximately 1–2 luminol units were conjugated on each β-CD. Further, the MALDI–TOF mass spectrum confirmed the conjugation of one luminol unit and three imidazole groups on each β-CD. In addition, a near-infrared AIE-active compound (PPV) was synthesized based on previously established procedures [41]. Synthesis of PPV was also characterized by ^1^H NMR, FT-IR, and liquid chromatography mass spectrometry (Additional file 1: Fig. S2). The AIE property of PPV was evaluated in mixtures of H_2_O and tetrahydrofuran (THF). Fluorescence emission intensities of PPV increased gradually as the volume ratio of H_2_O to THF changed from 20 to 90%, concomitant with a slight red shift of the maximum emission wavelength (Fig. 2A). In the presence of ClO^−^ (that can be produced by neutrophils via the MPO–H_2_O_2_–Cl^−^ system), LCD showed notable luminescence (Fig. 2B), with the maximum emission wavelength at 433 nm, which is consistent with the luminescence profile of free luminol (Additional file 1: Fig. S3). Of note, there is a considerable spectral overlap between the absorption spectrum of PPV and the luminescence spectrum of LCD, thereby enabling intermolecular CRET from LCD (a donor) to the AIE compound (an acceptor) [19]. In line with this result, we observed self-luminescence due to PPV (with the emission peak around 610 nm) in a mixture solution containing both LCD and PPV in the presence of ClO^−^ (Fig. 2C), indicating a good CRET effect between LCD and PPV.

To engineer self-illuminating nanoparticles with long-wavelength luminescence, PPV-loaded LCD nanoparticles were prepared by nanoprecipitation. By varying the weight ratio of PPV/LCD, LCD-derived nanoparticles with various contents of PPV were obtained (Additional file 1: Fig. S4). In view of desirable physicochemical properties of nanoparticles fabricated at the PPV/LCD weight ratio of 0.2/50 (defined as LAD NPs), they were used in the subsequent experiments. Characterization by transmission electron microscopy (TEM) indicated that LAD NPs displayed a spherical shape (Fig. 2D), showing a mean diameter of approximately 227 ± 15 nm. Dynamic light scattering (DLS) measurement revealed a relatively narrow size distribution of LAD NPs, with polydispersity of 0.12 (Fig. 2E). The mean diameter measured by DLS using five different batches of LAD NPs was 230 ± 10 nm. The ζ-potential value of LAD NPs was − 31.3 mV in deionized water.

### In vitro luminescence properties of LAD NPs

**Fig. 3:**
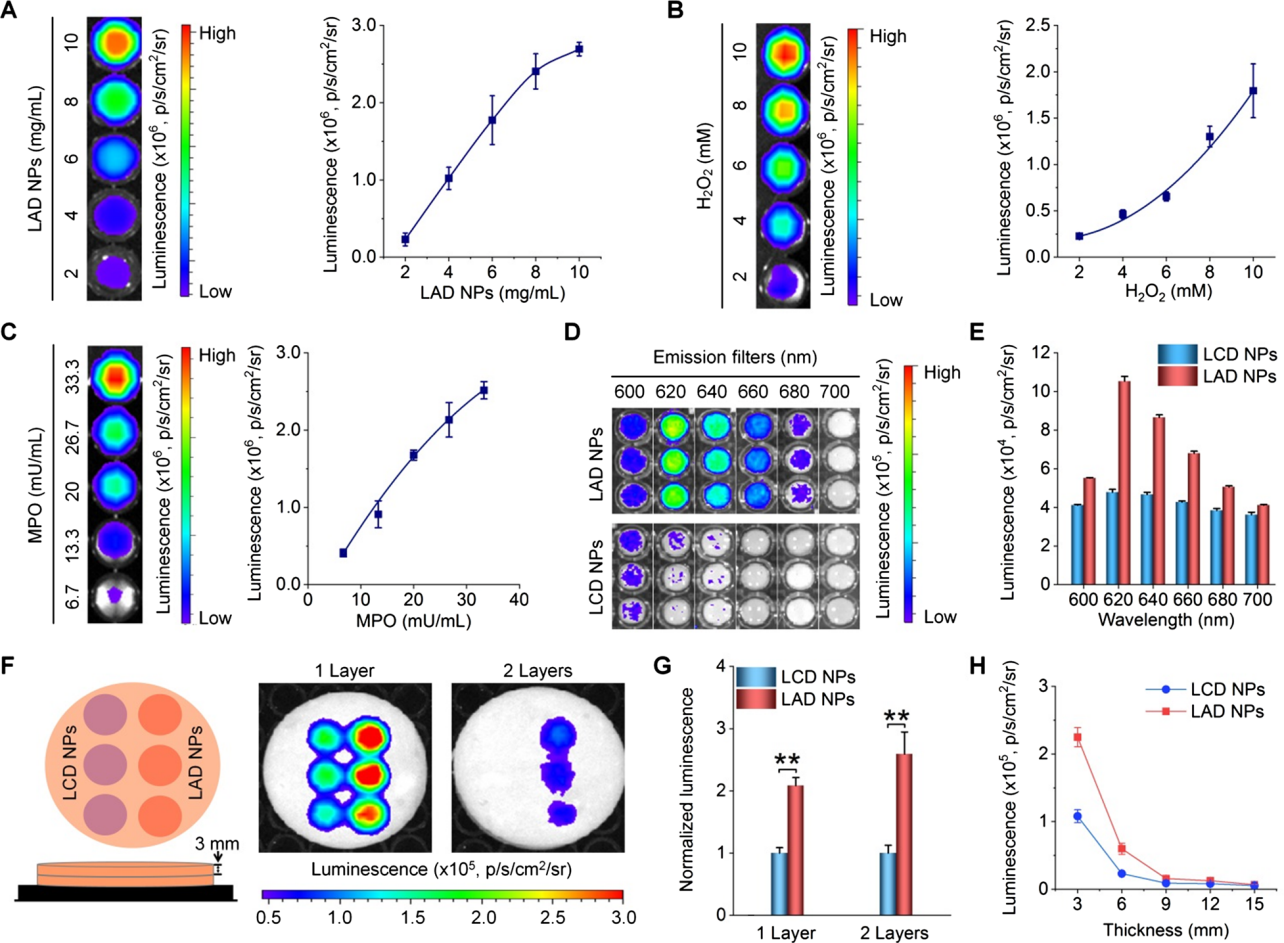
Luminescent properties and tissue penetration ability of LAD NPs. **A** Dose-dependent luminescent intensities of LAD NPs at 80 mM H_2_O_2_. **B** Effects of H_2_O_2_ levels on luminescent signals of 20 mg/mL LAD NPs. **C** MPO-dependent luminescence profiles of 20 mg/mL LAD NPs at 5 mM H_2_O_2_. In all cases, the left panels show luminescence images, while the right panels indicate quantitative data. **D**, **E** Typical luminescent images (**D**) and corresponding quantitative data of the same concentration of LAD NPs and LCD NPs at 80 mM H_2_O_2_ in the presence of emission filters of different wavelengths. **F**, **G** Luminescence images (**F**) and quantitative results (**G**) of the same concentration of LAD NPs and LCD NPs (at 15 mg/mL) in a black 96-well plate covered by 1 or 2 layers of 3-mm pork ham slices upon incubation with 80 mM H_2_O_2_. **H** Quantified luminescence intensities of LCD NPs or LAD NPs at 80 mM H_2_O_2_ and in the presence of different thicknesses of ham slices. Data are presented means ± SD (n = 3). **P < 0.01

As well documented, luminol is a chemiluminescent agent that can be oxidized by reactive oxygen species (ROS), such as hydrogen peroxide (H_2_O_2_) and hypochlorite (ClO^−^), to generate an aminophthalate ion [26, 29, 30]. Blue chemiluminescence (with the maximal emission at 440 nm) will be emitted, when this high energy intermediate (at an excited state) returns to its ground state by losing energy [29, 42]. Of note, the luminescence intensity can be considerably enhanced by myeloperoxidase (MPO), a peroxidase mainly expressed in neutrophils, largely by generating a strong oxidizing anion ClO^−^ via the MPO–H_2_O_2_–Cl^−^ system [43]. Accordingly, the effects of H_2_O_2_, MPO, and ClO^−^ on luminescence profiles of LAD NPs were examined. For engineered LAD NPs, spectrometric measurement revealed notable luminescence with a considerable CRET effect upon incubation with ClO^−^ (Fig. 2F). Also, quantification by an ultra-weak luminescence analyzer indicated that luminescence of LAD NPs was closely related to the concentration of LAD NPs and H_2_O_2_ (Fig. 2G, H). Meanwhile, sustained luminescence signals were detected for LAD NPs during incubation with H_2_O_2_ (Fig. 2I). We further examined luminescence properties of LAD NPs by imaging under different conditions, since this modality will be used for in vivo studies. Luminescence imaging indicated that the luminescence intensity of LAD NPs was dependent on the nanoprobe concentration at a defined level of H_2_O_2_ (Fig. 3A). Also, luminescence signals of LAD NPs were closely related to the H_2_O_2_ level (Fig. 3B), with higher levels of H_2_O_2_ affording stronger luminescence intensities. These results are consistent with those based on the luminescence analyzer. At low levels of H_2_O_2_ relevant to its content in activated neutrophils (~ 65 µM) [44], we found the similar luminescence behavior (Additional file 1: Fig. S5). In addition, the luminescence performance of LAD NPs depended on MPO (Fig. 3C). It is worth noting that MPO-responsive luminescence is a prerequisite for neutrophil imaging, since MPO is generally expressed by activated neutrophils [45].

Then we examined the CRET effect of LAD NPs by luminescence imaging, using nanoparticles based on LCD alone (i.e., LCD NPs) as a control nanoprobe (Additional file 1: Fig. S6). After applying emission filters of different wavelengths, luminescence imaging indicated that LCD NPs showed considerably weak luminescent signals at wavelengths larger than 600 nm upon incubation with H_2_O_2_ (Fig. 3D, E). By contrast, significantly higher luminescence intensities were detected for LAD NPs under the same conditions, with the luminescence peak at about 620 nm. This is well consistent with the result based on spectrometry measurement (Fig. 2F). Accordingly, these results confirmed the CRET effect between the luminol unit of LCD and PPV in LAD NPs, thereby emitting self-luminescence with notably long wavelengths. It should be emphasized that LAD NPs obtained from different batches showed no significant differences in the luminescence signal and CRET effect (Additional file 1: Fig. S7).

Further, we compared the tissue penetration capability of LAD NPs with LCD NPs by luminescence imaging in the presence of ham slices of different thicknesses. Whereas H_2_O_2_-triggered luminescence signals of both LCD NPs and LAD NPs notably decreased after various layers of ham slices were applied (Fig. 3F, G), LAD NPs displayed significantly stronger luminescence intensities when the tissue-simulating thickness was below 9 mm (Fig. 3H). This tissue penetrating result agrees with the long-wavelength luminescence performance of LAD NPs.

### In vitro luminescence imaging of neutrophils

**Fig. 4:**
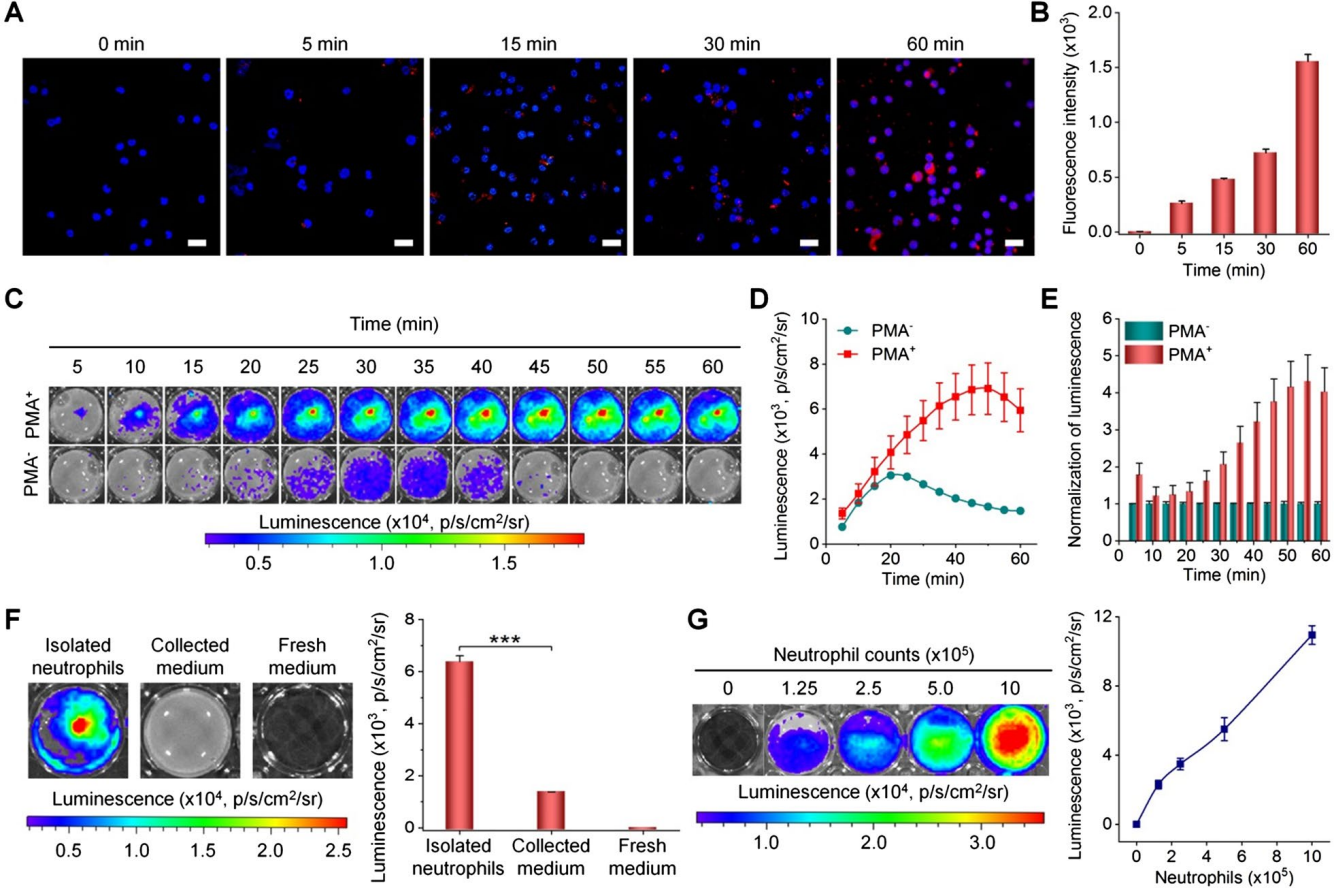
In vitro luminescence imaging of neutrophils with LAD NPs. **A**, **B** Confocal microscopy images (**A**) and flow cytometric analysis (**B**) showing time-dependent uptake of LAD NPs in peritoneal neutrophils. Nuclei were stained with DAPI. Scale bars, 20 μm. **C**, **D** Typical time-resolved images (**C**) and quantitative analysis (**D**) of luminescence signals in neutrophils treated with LAD NPs. Neutrophils (5 × 10^5^ cells per well) with (PMA^+^) or without (PMA^−^) stimulation with phorbol 12-myristate 13-acetate for 1 h were incubated with 3 mg/mL LAD NPs, followed by imaging at predetermined time points. **E** Normalized luminescence intensities in neutrophils. **F** Quantification of intracellular or extracellular luminescence intensities after neutrophils were incubated with 3 mg/mL LAD NPs for 1 h. **G** Luminescence intensities of different numbers of neutrophils immediately after incubation with 3 mg/mL LAD NPs. In both cases, the left panels show representative luminescence images, while the right panels denote quantitative data. Data are expressed as means ± SD (n = 3). ***P < 0.001

Neutrophils play an important role in tumor metastasis, the early recruitment of neutrophils in tumor metastases provides a comfortable microenvironment for metastasis [17, 45]. Based on the desirable luminescence performance, we conducted in vitro luminescence imaging studies in mouse neutrophils. Both confocal microscopic observation and flow cytometric quantification revealed rapid and time-dependent cellular uptake of LAD NPs by neutrophils (Fig. 4A, B). Then different inhibitors, including amiloride, nocodazole, chlorpromazine, genistein, and sodium azide were used to explore pathways dominating phagocytosis of LAD NPs by neutrophils. Of note, amiloride can inhibit micropinocytosis [46], while nocodazole may suppress F-actin polymerization and induce microtubule depolymerization, thereby attenuating endocytosis [47]. On the other hand, chlorpromazine and genistein are inhibitors of clathrin- and caveolae-mediated endocytosis, respectively [48, 49]. Sodium azide can attenuate energy-dependent internalization by depleting cellular ATP [50]. Flow cytometric analysis showed that cellular uptake of LAD NPs in neutrophils was significantly reduced by nocodazole, chlorpromazine, genistein, and sodium azide, but not by amiloride (Additional file 1: Fig. S8). These results substantiated that LAD NPs can be endocytosed by neutrophils through various pathways, involving caveolae/clathrin-mediated and ATP-dependent processes. This is consistent with the previous finding that neutrophils can internalize different NPs by endocytosis [51, 52].

Subsequently, in vitro luminescence imaging of neutrophils was examined. Upon incubation with LAD NPs, phorbol 12-myristate 13-acetate (PMA)-stimulated neutrophils showed significantly higher luminescence than unstimulated cells, in particular after 15 min of incubation (Fig. 4C–E). Moreover, strong luminescence signals of LAD NPs in PMA-activated neutrophils can sustain for about 1 h. Further, we isolated neutrophils and the culture medium after they were incubated with LAD NPs for 1 h. Luminescence imaging revealed the much stronger luminescent signal in neutrophils than that of the collected medium (Fig. 4F). This result suggested that luminescence signals are mainly generated in neutrophils upon incubation with LAD NPs. In addition, luminescence intensities of LAD NPs in neutrophils were positively correlated with the nanoprobe dose and neutrophil counts (Additional file 1: Fig. S9 and Fig. 4G). These results demonstrated that LAD NPs can be applied as an effective luminescent probe for imaging inflammation by illuminating activated neutrophils. Since the stability of LAD NPs in neutrophils for a certain time period is crucial to ensure the aggregated state of PPV, we detected changes in the particle size of LAD NPs after incubation with PBS or neutrophil lysates for different periods of time. It was found that the mean diameter of LAD NPs only slightly decreased after incubation with neutrophil lysates for 30 min (Additional file 1: Fig. S10). Therefore, LAD NPs are able to maintain their shape and size within a defined time period after endocytosis in neutrophils, thereby ensuring the aggregated state of PPV and CRET effect.

### In vivo luminescence imaging of lung metastasis via LAD NPs in a mouse model of breast cancer

**Fig. 5:**
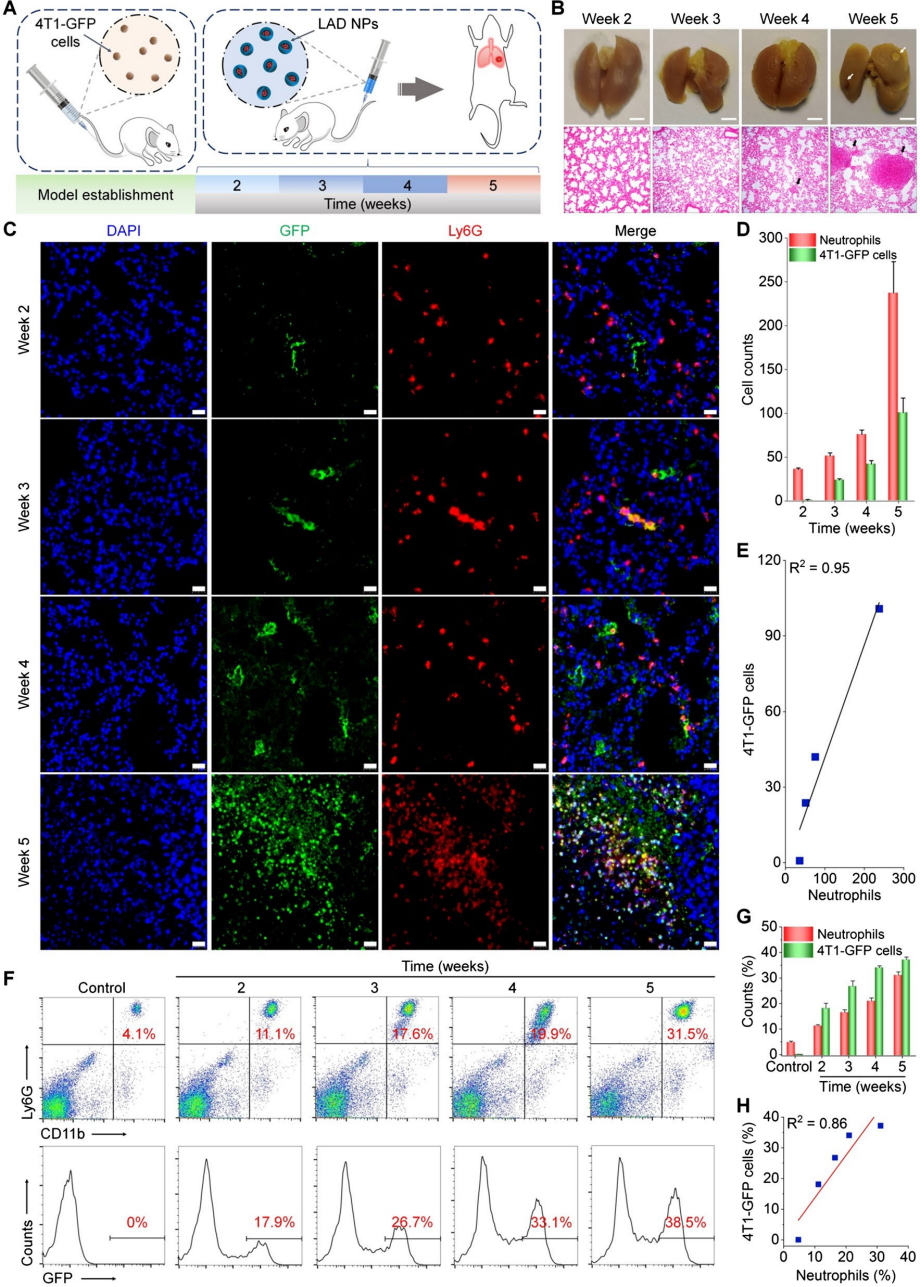
Correlation analyses of neutrophil and tumor cell counts in the lungs of mice subjected to intravenous inoculation with 4T1-GFP tumor cells. **A** Schematic illustration of experimental regimens. **B** Digital photos (upper) and microscopic images of H&E-stained histological sections of lungs (lower) at different time points after intravenous inoculation of 4T1-GFP tumor cells. Scale bars, 2 mm. Both white and black arrows indicate metastatic pulmonary nodules. **C** Immunofluorescence analyses of GFP-positive tumor cells (green) and Ly6G-positive neutrophils (red) in lung tissues of mice. Scale bars, 20 μm. **D** Quantitative analysis of the counts of 4T1-GFP tumor cells and neutrophils. **E** Correlation analysis of tumor cell and neutrophil counts in the lungs. **F** Representative flow cytometric profiles showing neutrophils (upper) and 4T1-GFP tumor cells (lower) in lung tissues. **G**, **H** Quantified cell populations of neutrophils and tumor cells by flow cytometry (**G**) and their correlation analysis (**H**). Data are expressed as means ± SD (**D**, n = 4; **G**, n = 5)

Subsequently, a mouse model of pulmonary metastasis was established by intravenous (i.v.) injection of mouse 4T1-GFP breast cancer cells in mice through the tail vein (Fig. 5A), to demonstrate our hypothesis that early diagnosis of lung metastases of cancers can be realized by luminescence imaging of pulmonary neutrophils. Previous studies have demonstrated the high incidence of lung metastasis after tail vein injection of tumor cells [53]. At different time points after i.v. injection of 4T1-GFP cells, both gross observation and examination on hematoxylin–eosin (H&E)-stained histological sections of lung tissues were conducted to monitor tumor progression. At weeks 4 and 5, we found the formation of pulmonary nodules that are typical for pulmonary metastases (Fig. 5B). Further, we interrogated the correlation between neutrophil infiltration and metastatic breast cancer in the lungs. To this end, changes in the numbers of neutrophils and cancer cells in the mouse lungs during pulmonary metastasis were analyzed by immunofluorescence. As early as 2 weeks after i.v. injection of cancer cells, immunofluorescence analysis revealed considerable filtration of neutrophils (Fig. 5C). At weeks 3, 4, and 5 post-injection of 4T1-GFP cells, further increased neutrophil counts were observed, showing a clearly time-dependent pattern (Fig. 5D). Whereas cancer cells in the lung could also be detected at week 2, their count was notably less than that of neutrophils. Moreover, quantitative analyses indicated good correlations between cancer cell and neutrophil counts (Fig. 5E). We further quantified neutrophil and tumor cell counts in the lung tissues of mice at different time points after inoculation with 4T1-GFP cells by flow cytometry, and the result also showed a good correlation between tumor cells and neutrophils (Fig. 5F–H). Together, these results implied that the colony formation at an early stage of pulmonary metastasis is accompanied with infiltration of relatively large numbers of neutrophils. Consequently, imaging or detection of pulmonary neutrophils can serve as an alternative and sensitive strategy for diagnosis of early pulmonary metastasis by amplifying relevant detection signals.

**Fig. 6:**
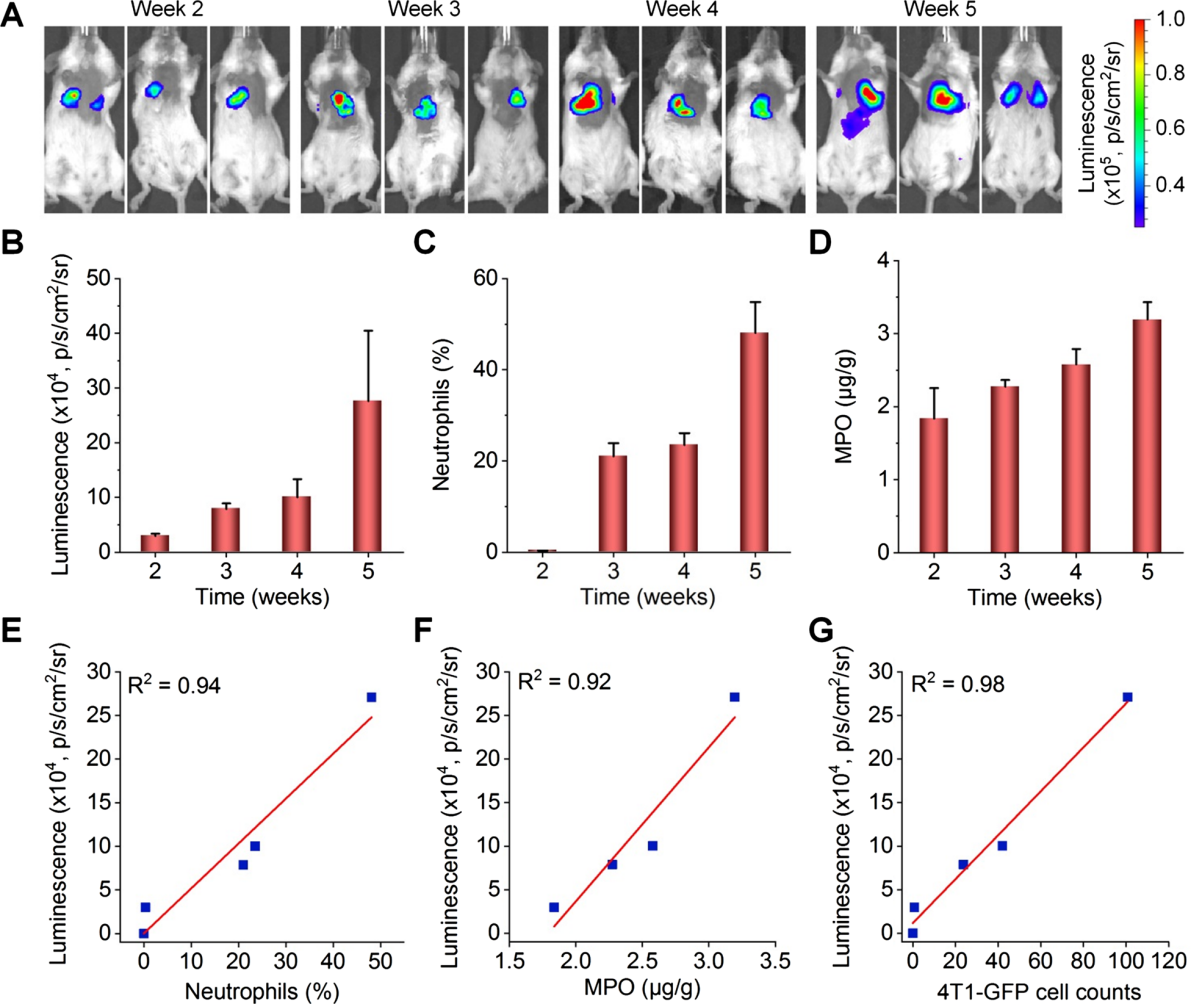
Luminescence imaging of pulmonary micrometastasis in mice with intravenously inoculated 4T1-GFP tumor cells by LAD NPs. **A** In vivo luminescence images showing pulmonary metastasis in mice. At various time points after i.v. inoculation of 4T1-GFP cells in mice, 3 mg LAD NPs was administered in each mouse by i.v. injection, immediately followed by luminescence imaging. **B** Quantitative analysis of pulmonary luminescence intensities in mice at various time points. **C** Percentages of neutrophils in bronchoalveolar lavage fluid (BALF) of mice after i.v. inoculation of 4T1-GFP cells for different time periods. **D** Quantified MPO levels in the lungs. **E**–**G** Correlation analyses of the luminescence intensity and the number of neutrophils in BALF (**E**), the MPO level in the lungs (**F**), or the 4T1-GFP cell counts in the lungs (**G**). Data are expressed as means ± SD (**B**, n = 3; **C**, n = 5; **D**, n = 6)

According to the above results, we performed luminescence imaging in mice at different time points after i.v. injection of 4T1 tumor cells, using LAD NPs as a self-illuminating nanosensor. Two weeks after i.v. inoculation, a notable luminescence signal due to LAD NPs was detected (Fig. 6A, B), and the luminescence intensity was further enhanced at weeks 3, 4, and 5. This changing profile of luminescence is similar to that of neutrophils in bronchoalveolar lavage fluid (BALF) of 4T1-GFP-inoculated mice, as quantified by flow cytometry (Fig. 6C). Likewise, the MPO level in the lungs was also gradually increased (Fig. 6D). In particular, we found good correlations between the luminescence intensity of LAD NPs and the neutrophil count, MPO level, or tumor cell count (Fig. 6E–G). Furthermore, flow cytometric quantification showed that LAD NPs were endocytosed by 42.2 ± 6.5% neutrophils in the lungs of mice at week 3 after i.v. inoculation of 4T1-GFP tumor cells (Additional file 1: Fig. S11). This suggested that luminescence signals in the lungs are mainly contributed by neutrophils, although the inflammatory niche can also lead to luminescence to a certain degree. Collectively, these results demonstrated that LAD NPs can function as an effective nanoprobe for luminescence imaging of neutrophil infiltration closely relevant to lung metastases of cancers.

### Luminescence imaging of breast cancer lung metastasis by neutrophil-targeting nanoparticles in mice

**Fig. 7:**
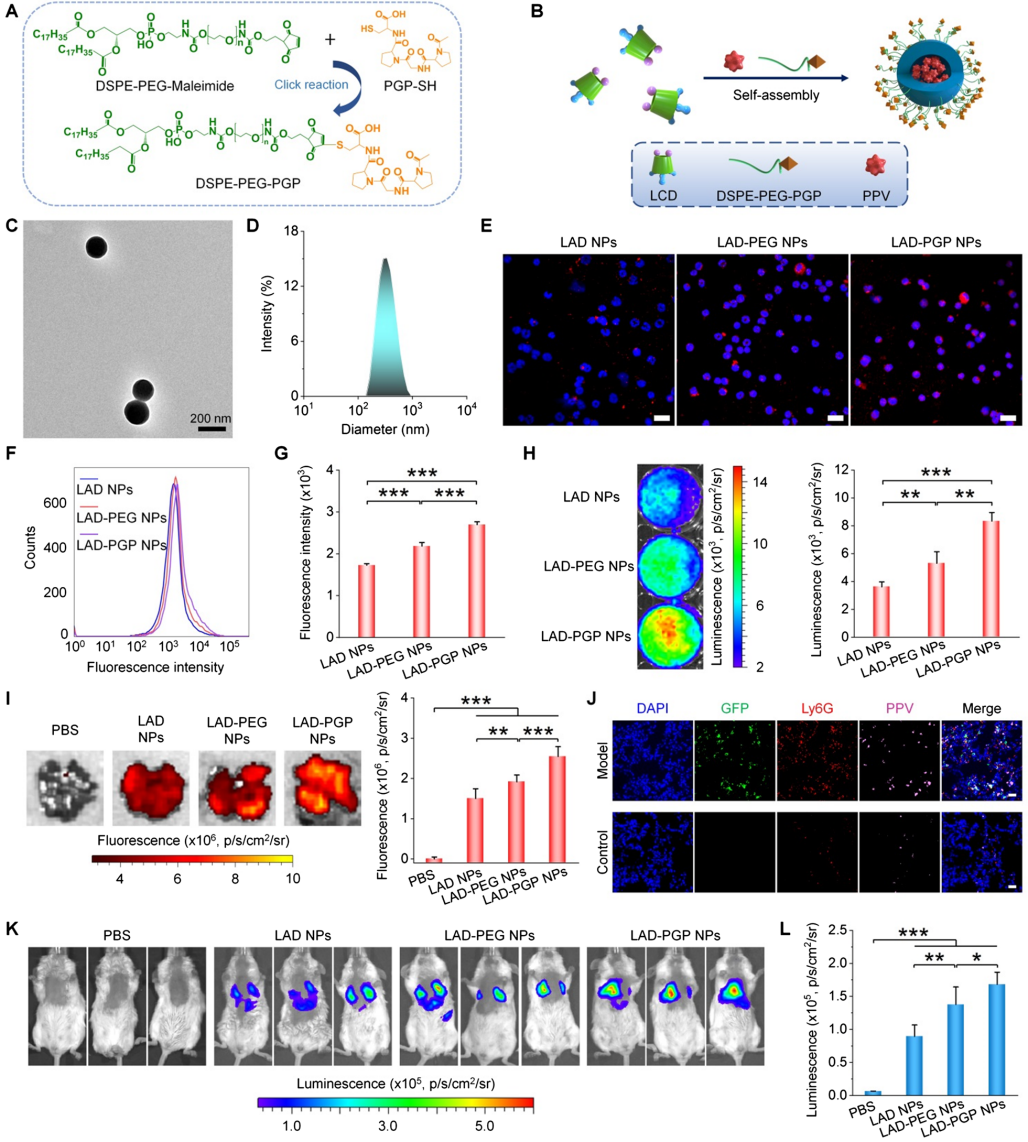
Engineering of a neutrophil-targeting self-luminescent nanoprobe for imaging of pulmonary micrometastasis in mice. **A** A scheme illustrating the synthesis of PGP peptide-conjugated DSPE-PEG (DSPE-PEG-PGP) capable of targeting neutrophils by binding to the CXCR2 receptor. **B** A sketch showing preparation of the neutrophil-targeting nanoprobe (LAD-PGP NPs). **C**, **D** A typical TEM image (**C**) and size distribution (**D**) of LAD-PGP NPs. **E** Fluorescence microscopic images of neutrophils at 1 h after incubation with the same dose of LAD NPs, LAD-PEG NPs, or LAD-PGP NPs. Nuclei were labeled with DAPI (blue). Scale bars, 20 μm. **F**, **G** Typical flow cytometry profiles (**F**) and quantitative data (**G**) of fluorescence intensities in neutrophils after 1 h of incubation with different NPs. **H** Luminescence images (left) and quantitative analysis (right) of PMA-stimulated neutrophils after treatment with different NPs. **I** Ex vivo fluorescence images (left) and quantified intensities (right) showing the accumulation of three NPs in lung tissues of mice at week 3 after i.v. inoculation of 4T1-GFP cells. Diseased mice injected with PBS served as a control. **J** Immunofluorescence images of lung tissue sections of healthy mice and diseased mice at week 3 after inoculation of 4T1-GFP cells. Lung tissues were isolated for analyses at 12 h after i.v. injection of 3 mg LAD-PGP NPs in each mouse. **K**, **L** Comparison of in vivo luminescence intensities in lung tissues after i.v. injection of 3 mg different nanoprobes in mice at week 3 after inoculation of 4T1-GFP cells. **K** Representative in vivo luminescence images. **L** Quantitative analysis of luminescence intensities. Data are expressed as means ± SD (**G**, **I**, **L**, n = 4; **H**, n = 3). *P < 0.05, **P < 0.01, ***P < 0.001

Based on the above promising findings, neutrophil-targeting luminescent NPs were engineered to further enhance the detection sensitivity. Previous studies have demonstrated the high binding affinity of *N*-acetyl proline–glycine–proline (PGP) peptide to the CXCR2 receptor on neutrophils [54, 55], while different PGP-decorated NPs showed notably increased cellular internazliation in neutrophils and enhanced targeting capability to specific tissues/organs by neutrophil-mediated transportation [56, 57]. PGP-conjugated DSPE-PEG was first synthesized by a thiol-maleimide click reaction (Fig. 7A). ^1^H NMR spectroscopy showed that characteristic peaks (at 6.69–6.72 ppm) of the maleimide group disappeared for the resulting product (Additional file 1: Fig. S12A, B), while proton signals (at 1.5–2.0 ppm) due to methyl and methylene groups in the PGP unit appeared. In addition, characteristic peaks corresponding to molecular weights of DSPE-PEG-PGP could be directly observed from the MALDI–TOF mass spectrum (Additional file 1: Fig. S12C). These results confirmed the successful synthesis of DSPE-PEG-PGP. Then DSPE-PEG-PGP was used to prepare PGP-decorated LAD NPs (i.e., LAD-PGP NPs) by a nanoprecipitation/self-assembly technique (Fig. 7B) [29]. TEM observation and DLS measurement showed spherical morphology and a narrow size distribution for the obtained LAD-PGP NPs (Fig. 7C, D), with the mean diameter of 232 ± 6 nm (quantified by DLS using five different batches of LAD-PGP NPs). Also, a control nanoprobe of PEGylated LAD NPs (i.e., LAD-PEG NPs) was produced using DSPE-PEG alone (Additional file 1: Fig. S13).

Observation via confocal microscopy and flow cytometric quantification indicated significantly enhanced cellular uptake of LAD-PGP NPs in neutrophils, compared to LAD-PEG NPs and LAD NPs (Fig. 7E–G). Correspondingly, neutrophils incubated with LAD-PGP NPs displayed significantly higher luminescence signals than those of neutrophils treated with LAD NPs or LAD-PEG NPs (Fig. 7H). After i.v. injection of different NPs in mice with simulated lung metastases of breast cancer, ex vivo imaging revealed the highest fluorescent signal (due to PPV) in the lungs isolated from LAD-PGP NP-treated mice (Fig. 7I). Notably, three groups treated with different NPs displayed comparable GFP fluorescence intensities, since the examined mice were inoculated with the same count of 4T1-GFP tumor cells (Additional file 1: Fig. S14). These results indicated that PGP functionalization can notably enhance lung targeting capability of LAD NPs. Further immunofluorescence analysis of lung sections revealed co-localization of LAD-PGP-NPs with neutrophils (Fig. 7J). Agreeing with the increased lung accumulation of LAD-PGP NPs, in vivo imaging showed the highest luminescence for LAD-PGP-NP-treated mice with 4T1 lung metastasis cancers (Fig. 7K, L). Collectively, these results demonstrated that luminescence imaging capacity of LAD NPs can be further amplified by neutrophil-mediated lung targeting via surface engineering with PGP.

**Fig. 8:**
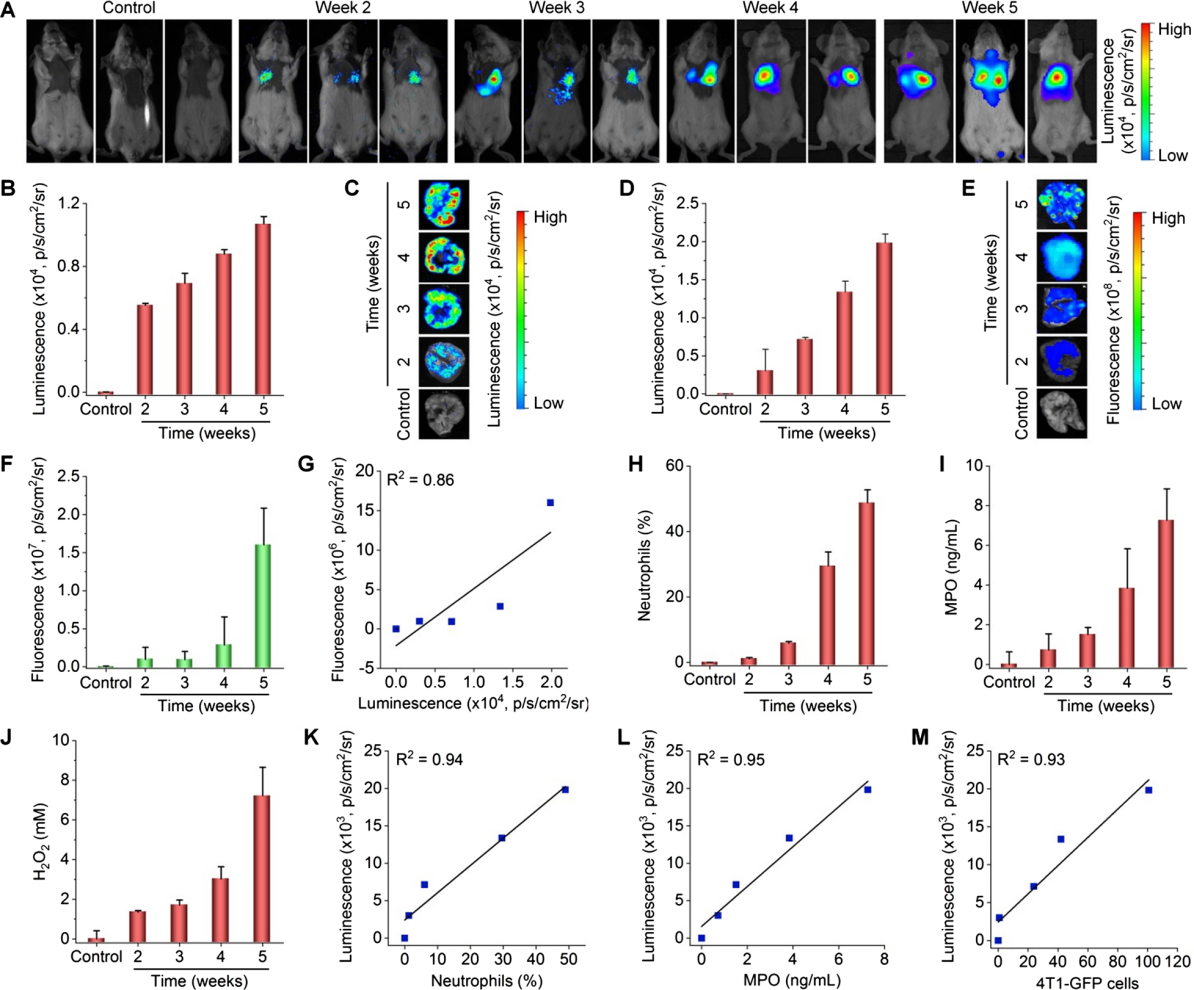
Luminescence imaging of early lung micrometastasis using LAD-PGP NPs in mice with inoculated 4T1-GFP cells. **A**, **B** Luminescence images (**A**) and quantitative analysis of luminescence intensities (**A**) of mice at different time periods after inoculation of 4T1-GFP cells. Images were acquired at 5 min after i.v. injection of 3 mg LAD-PGP NPs in each mouse. **C**, **D** Ex vivo luminescence images (**C**) and quantified intensities (**D**) for lung tissues isolated at different time points. **E**, **F** Ex vivo images (**E**) and quantitative analysis (**F**) of fluorescence intensities in isolated lung tissues. **G** Analysis of the correlation between GFP fluorescence and LAD-PGP NPs luminescence intensities in lung tissues. **H**–**J** Quantified neutrophil counts (**H**), MPO levels (**I**), and H_2_O_2_ concentrations (**J**) in BALF from mice at different time points after inoculation with 4T1-GFP cells. **K**–**M** Correlations between in vivo luminescence intensities of LAD-PGP NPs and neutrophil counts (**K**), MPO levels (**L**), or tumor cell counts (**M**). Data are expressed as mean ± SD (**B**, **D**, **F**, n = 3; **H**–**J**, n = 4)

Further, the lung metastasis process was monitored by luminescence imaging using LAD-PGP NPs. At weeks 2, 3, 4, and 5 after i.v. inoculation of 4T1-GFP cells, in vivo imaging of mice treated with LAD-PGP NPs revealed clearly time-dependent luminescence intensities (Fig. 8A, B). A similar changing profile of luminescent signals could be found by ex vivo luminescence images of isolated lungs (Fig. 8C, D). Correspondingly, ex vivo fluorescence imaging of isolated lungs indicated gradually enhanced GFP fluorescence signals at the examined time points (Fig. 8E, F). Also, the presence of GFP fluorescence in excised lungs at week 2 suggested the colony formation of tumor cells in the lungs at this time point. However, luminescence intensities of LAD-PGP NPs did not linearly correlate with GFP fluorescence signals in the lungs (Fig. 8G), likely due to considerable tissue absorption of short-wavelength GFP fluorescence. In support of this point, in vivo fluorescence imaging only afforded very poor results due to interference of autofluorescence signals (Additional file 1: Fig. S15). Consistently, notably increased neutrophil counts as well as levels of MPO and H_2_O_2_ in BALF were also detected at the examined time points (Fig. 8H–J and Additional file 1: Fig. S16). In particular, linear correlations were found between the luminescence intensity and the neutrophil count, MPO level, or H_2_O_2_ concentration (Fig. 8K, L and Additional file 1: Fig. S17). Of note, we also found that, in this case, luminescence signals of LAD-PGP NPs well correlated with tumor cell counts in the lungs (Fig. 8M). Consequently, the targeting LAD-PGP NPs can be used as a more effective luminescent nanoprobe for early detection of cancer metastasis by imaging the pre-metastatic niche in the lungs that is characterized with the colony formation by a few tumor cells, concomitant with neutrophil infiltration and oxidative stress.

### Comparison of the lung metastasis imaging performance of LAD-PGP NPs with clinically used imaging modalities

**Fig. 9:**
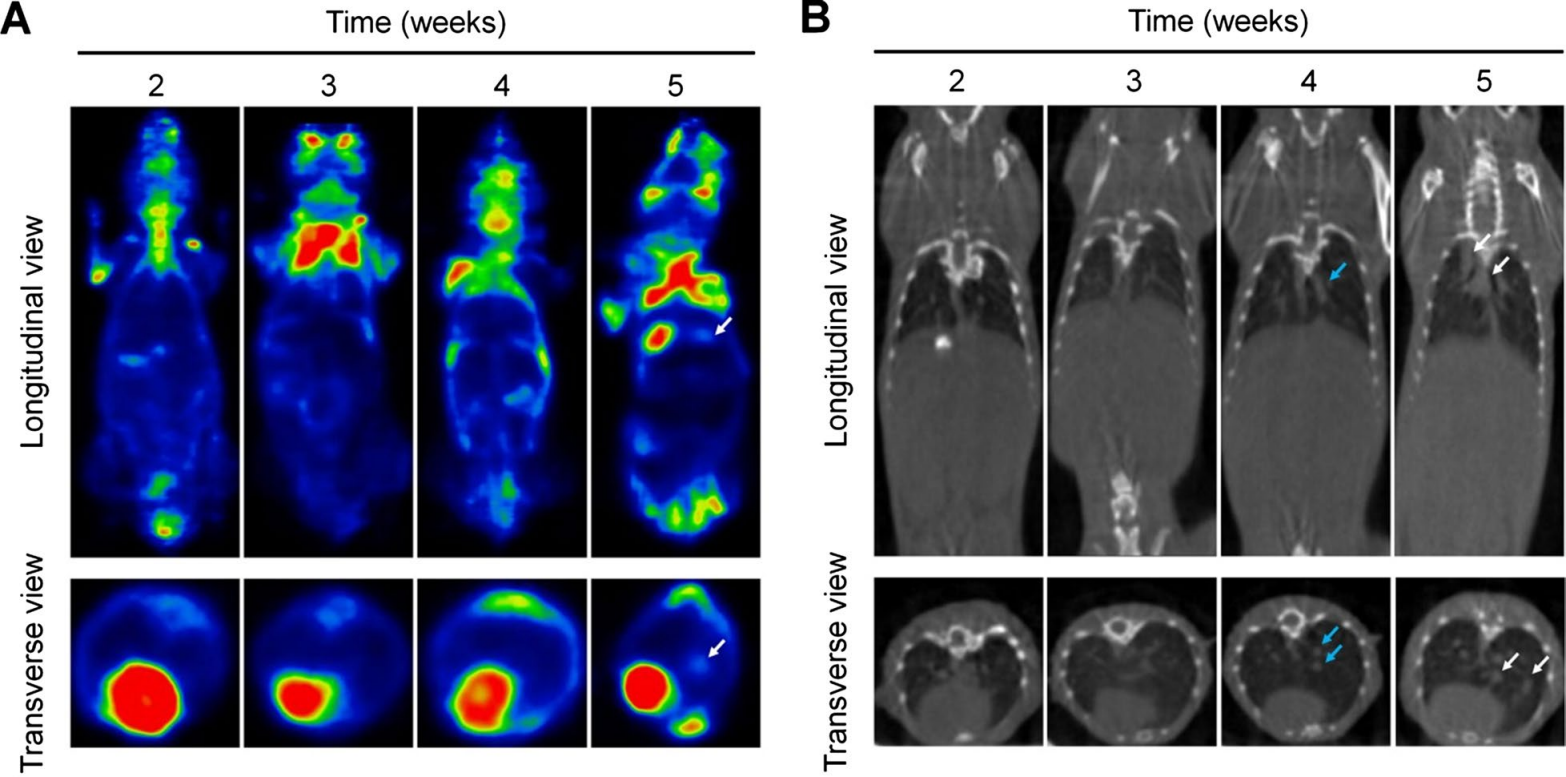
PET and CT imaging of lung metastasis in mice. **A**, **B** Representative PET (**A**) and CT (**B**) images of mice at different time periods after inoculation of 4T1-GFP cells. Metastatic pulmonary nodules are indicated with white arrows, while blue arrows indicate lung consolidation and enlarged lymph nodes. For PET imaging, ^18^F-FDG (200–300 µCi in each mouse) was used as a radiotracer, with the exposure time of 20 min

Currently, positron emission tomography (PET) and/or computed tomography (CT) have been generally employed for detection of pulmonary metastases of different cancers in the clinic [58, 59]. However, precise and sensitive diagnosis of early metastasis to the lungs remains highly challenging, since the small size colony formed by tumor cells at an early stage of tumor metastasis cannot be accurately detected by these clinically used strategies. Therefore, we compared diagnostic effects of our nanoprobe LAD-PGP NPs with PET and CT. As mentioned above, the lung metastasis model in mice was established by i.v. inoculation of 4T1-GFP cells. PET imaging using ^18^F-fluorodeoxyglucose (^18^F-FDG) as a PET radiotracer showed concentrated radionuclide molecules in the lungs at week 5 (Fig. 9A), while no significant signals were detected at weeks 2, 3, and 4. This may be due to the low metabolism of tumor micrometastases at early stages, since no obvious nodules were formed. On the other hand, CT imaging results only revealed the enlargement of hilar lymph nodes and a small area of consolidation at week 4 (Fig. 9B). Clear lung metastasis could be scanned at week 5. Consequently, PET/CT imaging can only detect large metastatic nodules in the lungs, but cannot monitor pre-metastatic niches and/or micrometastases.

Although some near infrared (NIR) fluorescent dyes, such as indocyanine green (ICG) and cyanine7.5, have been broadly used for in vivo fluorescence imaging of tumors and optical image-guided cancer surgery [60], due to their deep tissue penetration capability. However, these NIR probes need to be excited by external irradiation to generate fluorescence. In addition, their fluorescence signals are mainly determined by the tissue/cellular distribution, other than pathophysiological microenvironments. Meanwhile, these types of NIR dyes used for tumor imaging and/or diagnosis are mostly based on the developed tumor nodules. By contrast, our neutrophil-targeting luminescent nanoprobe is able to image pre-metastatic niches and micrometastases by illuminating the tumor-associated inflammatory microenvironment. Accordingly, luminescent imaging based on long-wavelength nanoprobes is a promising modality for early diagnosis or prediction of lung metastases.

### Discrimination of neutrophil infiltration due to metastasis and pulmonary inflammation

For potential applications of luminescence imaging of early lung metastases by our nanoprobes, one of critical concerns lies in possible false positive results due to the neutrophil infiltration causing by pulmonary infections. As well documented, a large number of neutrophils infiltrate the lungs during the acute phase of infections [17]. To distinguish pulmonary inflammatory diseases (such as acute injury and infection) from lung metastasis, we established mouse models of acute lung injury by intranasal inhalation of lipopolysaccharide (LPS) and lung infection by intratracheal injection of Klebsiella pneumoniae (Additional file 1: Fig. S18A) [61–64]. For both mouse models, strong luminescence signals were detected in the lungs by in vivo imaging, at 5 min after i.v. injection of the active targeting nanoprobe LAD-PGP NPs (Additional file 1: Fig. S18B, C). This is in line with neutrophil infiltration in the inflamed or infected lungs [64].

Nevertheless, for infected mice, blood routine examination revealed significantly higher neutrophil counts in the peripheral blood, compared to those of saline-treated mice (Additional file 1: Fig. S19). By contrast, mice with 4T1 tumors in the lungs showed no significantly increased blood neutrophils, when compared with healthy mice. In addition, the blood neutrophil count of the infection group was significantly higher than that of the lung metastasis group. These preliminary results indicated that lung infections are accompanied with significantly increased levels of neutrophils in peripheral blood, while lung metastasis of cancers does not cause notable changes in blood neutrophils. Therefore, a typical blood routine test in combination with the proposed luminescence imaging modality can be performed to confirm lung metastases of breast cancers. On the other hand, the above results also suggested that our targeting nanoprobe can be used for luminescence imaging of acute inflammation in the lung.

### Safety evaluations of LAD NPs

Finally, safety profiles of LAD NPs were preliminarily evaluated by in vitro and in vivo studies. After different doses of LAD NPs were separately incubated with 4T1 cancer cells and peritoneal neutrophils for 12 h, high cell viability was detected for both cells (Additional file 1: Fig. S20). Even at 500 µg/mL of LAD NPs, no significant cytotoxicity was found, demonstrating extremely low cytotoxicity of LAD NPs in both normal and tumor cells.

Subsequently, acute toxicity of LAD NPs was evaluated in mice after a single i.v. injection at 500 and 1000 mg/kg. For all LAD NPs-treated mice, they had no abnormal daily behaviors and no significant weight changes (Additional file 1: Fig. S21). On day 15 after treatment, animals were euthanized, and no significant abnormalities in the organ index of major organs were observed (Additional file 1: Fig. S22). Complete blood count revealed no abnormal variations in the numbers of white blood cells, red blood cells, and platelets as well as the level of hemoglobin (Additional file 1: Fig. S23). The levels of typical biomarkers relevant to functions of the liver and kidneys were normal for LAD NPs-treated mice (Additional file 1: Fig. S24). Further examination on H&E-stained histological sections indicated no obvious inflammatory infiltration, cell necrosis, or tissue injury in the heart, liver, spleen, lung, and kidney resected from mice treated with two doses of LAD NPs (Additional file 1: Fig. S25). These preliminary results suggested that LAD NPs can serve as a safe nanoprobe for luminescence imaging of early lung metastases.

## Conclusions

In summary, we proposed and demonstrated a new and promising modality for early diagnosis of lung metastasis of breast cancers, according to pathological features of pre-metastatic niches in the lungs, which are characterized with the considerable infiltration of neutrophils and overexpression of oxidative mediators. To this end, a luminescent nanosensor (i.e., LAD NPs) with long emission wavelengths was engineered by combination of a luminol-functionalized cyclodextrin self-luminescent material and an AIEgen, taking advantage of the CRET effect. LAD NPs can illuminate activated neutrophils, due to the MPO/ROS-responsive self-luminescence performance. In a mouse model of lung metastasis of breast cancer, LAD NPs afforded desirable luminescent signals closely relevant to the colony formation by cancer cells at an early stage of lung metastasis, resulting from pre-metastatic microenvironment-mediated signal amplification. By functionalizing LAD NPs with a neutrophil-targeting peptide, the obtained active targeting nanoprobe showed notably improved imaging capability for early detection of pulmonary metastasis. Furthermore, our targeting nanoprobe-based luminescence imaging strategy remarkably outperformed PET/CT imaging modalities in the examined mouse model, with respect to early prediction of lung metastatic nodules. Also, our preliminary tests demonstrated a good safety profile of LAD NPs for diagnosis by i.v. injection.

## Supplementary Information

**Additional file 1: Materials and Methods. Scheme S1.** Synthesis of PPV. **Fig. S1**. Structural characterization of a luminescent material LCD. **Fig. S2**. Characterization of PPV. **Fig. S3.** The luminescence spectrum of luminol in the presence of hypochlorite. **Fig. S4**. TEM images of LAD NPs containing various contents of PPV. **Fig. S5**. Luminescence profiles of LAD NPs at pathophysiological concentrations of H_2_O_2_. **Fig. S6**. A sketch showing preparation of LCD nanoparticles (LCD NPs) by nanoprecipitation. **Fig. S7**. Comparison of luminescence intensities of LAD NPs based on three different batches in the presence of ClO^-^. **Fig. S8**. The effects of different inhibitors on cellular uptake of LAD NPs in neutrophils. **Fig. S9**. Dose-dependent luminescence of LAD NPs in peritoneal neutrophils. **Fig. S10**. Changes in the mean diameter of LAD NPs after incubation with PBS or neutrophil lysates. **Fig. S11**. Flow cytometric analysis of the distribution of LAD NPs in neutrophils in the lung tissue of mice at week 3 after i.v. inoculation of 4T1-GFP tumor cells. **Fig. S12**. Spectroscopy characterization of PGP-conjugated DSPE-PEG (DSPE-PEG-PGP). **Fig. S13**. Schematic illustration of preparation of PPV-loaded PEGylated LCD NPs (LAD-PEG NPs) by a nanoprecipitation/self-assembly method. **Fig. S14**. Ex vivo imaging of GFP fluorescence intensities in lung tissues of mice at week 3 after i.v. inoculation of 4T1-GFP tumor cells. **Fig. S15**. In vivo fluorescence images of mice inoculated with 4T1-GFP cells by i.v. injection. **Fig. S16**. Flow cytometric profiles showing neutrophil counts in bronchoalveolar lavage fluid (BALF) from mice with or without inoculation of 4T1 cells. **Fig. S17**. Analysis of correlation between the luminescence intensity and the H_2_O_2_ concentration in BALF. **Fig. S18**. Luminescence imaging of acute inflammation in the lungs with LAD-PGP NPs. **Fig. S19**. Comparison of peripheral blood neutrophils in mice with lung metastasis or lung infection. **Fig. S20**. Cytotoxicity evaluation of LAD NPs. **Fig. S21**. Changes in mouse body weight after a single i.v. administration of LAD NPs at 500 or 1000 mg/kg. **Fig. S22**. The organ index of typical major organs at day 15 after treatment with LAD NPs. **Fig. S23**. The blood levels of WBC, RBC, PLT, and HGB at day 15 after treatment with various doses of LAD NPs. **Fig. S24**. Serum levels of ALT, AST, UREA, and CREA at day 15 after treatment with various doses of LAD NPs. **Fig. S25**. H&E-stained pathological sections of typical major organs.

## Data Availability

The authors confirm that the data supporting the findings of this study are available within the article.
