# Supplementary material for: Early diagnosis of breast cancer lung metastasis by nanoprobe-based luminescence imaging of the pre-metastatic niche
Source: J Nanobiotechnology. 2022 Mar 15;20:134. doi: 10.1186/s12951-022-01346-4 (PMC8922882; doi:10.1186/s12951-022-01346-4)
Supplement: Supplementary file 1 — Additional file 1: Materials and Methods. Scheme S1. Synthesis of PPV. Fig. S1. Structural characterization of a luminescent material LCD. Fig. S2. Characterization of PPV. Fig. S3. The luminescence spectrum of luminol in the presence of hypochlorite. Fig. S4. TEM images of LAD NPs containing various contents of PPV. Fig. S5. Luminescence profiles of LAD NPs at pathophysiological concentrations of H2O2. Fig. S6. A sketch showing preparation of LCD nanoparticles (LCD NPs) by nanoprecipitation. Fig. S7. Comparison of luminescence intensities of LAD NPs based on three different batches in the presence of ClO-. Fig. S8. The effects of different inhibitors on cellular uptake of LAD NPs in neutrophils. Fig. S9. Dose-dependent luminescence of LAD NPs in peritoneal neutrophils. Fig. S10. Changes in the mean diameter of LAD NPs after incubation with PBS or neutrophil lysates. Fig. S11. Flow cytometric analysis of the distribution of LAD NPs in neutrophils in the lung tissue of mice at week 3 after i.v. inoculation of 4T1-GFP tumor cells. Fig. S12. Spectroscopy characterization of PGP-conjugated DSPE-PEG (DSPE-PEG-PGP). Fig. S13. Schematic illustration of preparation of PPV-loaded PEGylated LCD NPs (LAD-PEG NPs) by a nanoprecipitation/self-assembly method. Fig. S14. Ex vivo imaging of GFP fluorescence intensities in lung tissues of mice at week 3 after i.v. inoculation of 4T1-GFP tumor cells. Fig. S15. In vivo fluorescence images of mice inoculated with 4T1-GFP cells by i.v. injection. Fig. S16. Flow cytometric profiles showing neutrophil counts in bronchoalveolar lavage fluid (BALF) from mice with or without inoculation of 4T1 cells. Fig. S17. Analysis of correlation between the luminescence intensity and the H2O2 concentration in BALF. Fig. S18. Luminescence imaging of acute inflammation in the lungs with LAD-PGP NPs. Fig. S19. Comparison of peripheral blood neutrophils in mice with lung metastasis or lung infection. Fig. S20. Cytotoxicity evaluation of LAD NPs. Fig. S21. C [file 12951_2022_1346_MOESM1_ESM.docx]

Additional Information

**Early diagnosis of breast cancer lung metastasis by nanoprobe-based luminescence imaging of the pre-metastatic niche**

Hanwen Zheng^1,2,3^, Chunsen Yuan^1^, Jiajun Cai^2^, Wendan Pu^2^, Peng Wu^2,4^, Chenwen Li^2^, Gang Li^2^, Yang Zhang^2^, Jianxiang Zhang^2,5,^*, Jiawei Guo^3,^* and Dingde Huang^1,^*

1. Department of Nuclear Medicine, Southwest Hospital, Third Military Medical University (Army Medical University), Chongqing 400038, China
2. Department of Pharmaceutics, College of Pharmacy, Third Military Medical University (Army Medical University), Chongqing 400038, China
3. Department of Pharmaceutical Analysis, College of Pharmacy, Third Military Medical University (Army Medical University), Chongqing 400038, China
4. College of Pharmacy and Medical Technology, Hanzhong Vocational and Technical College, Hanzhong, Shaanxi 723000, China
5. State Key Laboratory of Trauma, Burn and Combined Injury, Third Military Medical University (Army Medical University), Chongqing 400038, China

**Corresponding authors:**

Jianxiang Zhang, PhD, Prof.

Department of Pharmaceutics

College of Pharmacy

Third Military Medical University (Army Medical University)

30 Gaotanyan Main Street, Chongqing 400038, China

E-mail: jxzhang1980@gmail.com, jxzhang@tmmu.edu.cn

Jiawei Guo, PhD, Prof.

Department of Pharmaceutical Analysis

College of Pharmacy

Third Military Medical University (Army Medical University)

30 Gaotanyan Main Street, Chongqing 400038, China

E-mail: breakhp@163.com

Dingde Huang, PhD, Prof.

Department of Nuclear Medicine

Southwest Hospital

Third Military Medical University (Army Medical University)

30 Gaotanyan Main Street, Chongqing 400038, China

E-mail: huangdingde@126.com

**Materials and Methods**

**Materials.** Luminol, luminol sodium, β-cyclodextrin (β-CD), 1,1'-carbonyldiimidazole (CDI), anhydrous dimethylformamide (DMF), phorbol 12-myristate 13-acetate (PMA), thioglycolate, and 4',6-diamidino-2-phenylindole (DAPI) were purchased from Sigma-Aldrich (USA). Myeloperoxidase (MPO) was obtained from BioVision, Inc. (California, USA). Fetal bovine serum (FBS) was obtained from Gibco (USA). Anti-mouse Ly6G antibody and mouse MPO antibody were purchased from BioLegend, Co. (San Diego, USA) and R&D Systems Inc. (Minneapolis, USA), respectively. 1,2-Distearoyl-sn-glycero-3-phosphoethanolamine-N-[maleimide-2000] (DSPE-PEG-Maleimide) was purchased from Nanosoft Polymers (Winston-Salem NC, USA). N-Acetyl Pro-Gly-Pro-Cys (PGP-SH) peptide was obtained from Sangon Biotech, Co. Ltd (Shanghai, China). The ELISA kit of MPO and Amplex Red Hydrogen Peroxide Assay Kit were purchased from Boster Biological Technology Co. Ltd (USA) and Thermo Fisher (USA), respectively. Anti-mouse APC-CD11b antibody and anti-mouse PE-Ly6G antibody were purchased from BD Biosciences (USA). GFP antibody and DK-GFP antibody were purchased from Abcam and Sigma-Aldrich (USA). Nocodazole, amiloride, chlorpromazine, genistein, and sodium azide were purchased from MCE (China). Liberase TM and DNase I (deoxyribonuclease I) were purchased from Roche and Sigma-Aldrich (USA), respectively. Red blood cell lysis buffer was obtained from Beyotime Biotechnology (China). Lipopolysaccharide (LPS) from Escherrichia Coli O111:B4 was purchased by Sigma-Aldrich (USA).

**Synthesis of a Luminescent β-Cyclodextrin (β-CD)** **Material.** Specifically, β-CD (0.68 g, 0.7 mmol) dried at 60°C in vacuum overnight and 1,1'-carbonyldiimidazole (CDI) (0.68 g, 4.2 mmol) were co-dissolved in 6 mL of anhydrous dimethylformamide (DMF). The solution was magnetically stirred at 20-25°C for 1.5 h, followed by precipitation in cold diethyl ether. The resulting CDI-activated β-CD (CDI-CD) was dissolved in anhydrous DMF (10 mL), into which luminol (0.318 g, 1.8 mmol) was added, followed by reaction at 20-25°C for additional 15 h under nitrogen. After precipitation from deionized water, luminol-conjugated β-CD (LCD) was collected by centrifugation and lyophilization.

**Synthesis of N-Acetyl Pro-Gly-Pro-Cys (PGP-SH) Peptide-Conjugated DSPE-PEG.** DSPE-PEG Maleimide (14 mg, 0.004 mmol) was dissolved in 0.01 M PBS (pH 7.4), into which 4 mg PGP-SH peptide (0.008 mmol) dissolved in PBS was added. The mixture was magnetically stirred at 20-25°C for 24 h, followed by dialysis (MWCO, 2000 Da) in deionized water. DSPE-PEG-PGP was harvested after lyophilization.

**Synthesis of an Aggregation-Induced Emission (AIE) Compound of 2,5-Bis(diphenylamino) tereph-thaldicarboxyalde-hyde (PPV).** PPV was synthesized according to the previously established method (Scheme S1) [1].

**Synthesis of Compound 1:** Dimethyl succinylosuccinate (DMSS, 5 g, 22 mmol) was placed into a round bottomed flask. Aniline (100 mL) was used in excess as a solvent and the reaction mixture was heated to 100°C for 2 h, during which a solid precipitate appeared in the reaction solution. Thin Layer Chromatography (TLC) analysis (CH_2_Cl_2_ : hexane = 1:1) showed the presence of DMSS. The reaction temperature was then increased to 120°C and stirred for additional 4 h. The reaction mixture was cooled, and EtOH was added. The product (**Compound 1**) was collected by vacuum filtration.

**Synthesis of Compound 2: Compound 1** (4 g, 10.5 mmol) was dissolved in CH_2_Cl_2_ and heated, during which the solution turned into dark red. TLC analysis (CH_2_Cl_2_ : hexane = 1:1) was used to monitor the reaction. Once all the starting material disappeared, CH_2_Cl_2_ was partially removed by rotary evaporation and replaced with EtOH. After all CH_2_Cl_2_ was removed, the red solid was collected by vacuum filtration and washed with EtOH until the product become colorless. The final product (**Compound 2**) was collected and dried to give a red solid powder.

**Synthesis of Compound 3: Compound 2** (3 g, 8.25 mmol), iodobenzene (200 mL, 134.25 mmol), K_2_CO_3_ (20 g, 10.5 mmol), and copper (0.105 g, 1.65 mmol) were placed in a round-bottomed flask under N_2_. The mixture was heated at 185°C for 24 h. TLC (CH_2_Cl_2_ : hexane = 1:1) indicated the completion of the reaction. Then the reaction mixture was cooled, and CH_2_Cl_2_ was added. After the reaction mixture was filtered and washed with CH_2_Cl_2_, the filtrate was concentrated to give a suspension. EtOH was added, and the resulting precipitate was collected by filtration and washed with EtOH. The final product was dried to give a bright yellow solid powder (**Compound 3**).

**Synthesis of Compound 4:** To a round-bottomed flask equipped with a reflux condenser, 20 mL of anhydrous THF was added and cooled to 0°C, into which LiAlH_4_ (6.4 g, 0.93 mmol) was added under N_2_ to form a suspension. **Compound 3** (40 g, 3.8 mmol) was added over 60 min. The mixture was warmed to room temperature for 15 min and then refluxed for 45 min. Then the reaction mixture was cooled to 0°C and quenched carefully with water. After stirring for 30 min, ether was added. The aqueous layer was extracted four times with ether. The combined organic phase was dried over Na_2_SO_4_ and concentrated to give a brown solid. The solid was recrystallized from toluene to give a yellow solid (**Compound 4**).

Finally, pyridinium chlorochromate (1 g, 2.1 mmol) and anhydrous CH_2_Cl_2_ (10 mL) were placed in a flask under N_2_. **Compound 4** was added gradually, during which time the mixture turned black. TLC (CH_2_Cl_2_ : hexane = 1:1) indicated the disappearance of the starting material after 90 min, while a new red spot appeared. The entire reaction mixture was filtered through a pad of Celite on a fritted funnel and washed thoroughly with CH_2_Cl_2_ until the washings were almost colorless. The filtrate was concentrated and purified by column chromatography (CH_2_Cl_2_ : hexane = 1:1) to give a pure product as a dark red crystalline solid powder (PPV). ^1^H NMR (600 MHz, CDCl_3_) δ (ppm): 10.12 (s, 2H), 7.60 (s, 2H), 7.27 (t, J = 7.8 Hz, 8H), 7.03 (t, J = 7.9 Hz, 12H).

**Materials Characterization.** Fourier-transform infrared (FT-IR) spectra were recorded on a PerkinElmer FT-IR spectrometer (100S, USA). UV-Vis spectroscopy was conducted on an ultraviolet spectrophotometer (TU-1901, Beijing Purkinje General instrument, China). ^1^H NMR spectra were acquired on a nuclear magnetic resonance (NMR) spectrometer operating at 600 MHz (DD2, Agilent). Matrix-assisted laser desorption/ionization time-of-flight (MALDI-TOF) mass spectrometry was conducted on an ultrafleXtreme TOF/TOF instrument (Bruker). Liquid chromatography mass spectrometry was performed using a Waters Acquity UPLC system (USA).

**The AIE Property of PPV.** PPV (0.1 mg) was separately dissolved in 1 mL of solvent mixture of water/THF with the water content varying from 20%, 40%, 60%, 80%, to 90%. Then fluorescence spectra of different samples were acquired using a fluorescence spectrophotometer (Hitachi, F-7000).

**The CRET Effect between LCD and PPV.** Sodium hypochlorite (100 mM) was added to 50 mg/mL LCD in DMF, and the emission spectrum was acquired. On the other hand, the excitation spectrum of PPV at 1 mg/mL in DMF was acquired by a fluorescence spectrophotometer, with an emission wavelength at 650 nm. In addition, the luminescence spectrum of luminol (0.5 mM) in Na_2_CO_3_ solution containing sodium hypochlorite (100 mM) was collected by a fiber optic spectrometer (AvaSpec-HS, Avantes, Netherlands).

In another experiment, 100 μL of 50 mg/mL LCD in DMF was mixed with 300 μL of 2 mg/mL PPV in DMF, into which 100 μL of 100 mM sodium hypochlorite was added. The luminescence spectrum of the mixed solution was acquired by a fiber optic spectrometer.

**Fabrication of LAD Nanoparticles (LAD NPs) by Nanoprecipitation.** In brief, 50 mg LCD and 0.2 mg PPV were dissolved in 2 mL of DMF, into which 10 mL of deionized water was added dropwise over 2 h with magnetic stirring at 20-25°C. NPs were collected after centrifugation at 16000g and washed with deionized water three times. To fabricate NPs containing different contents of PPV, different amounts of PPV were dissolved in DMF before deionized water being added.

**Preparation of PGP-Modified LAD NPs.** To fabricate PGP-modified LAD NPs (i.e., LAD-PGP NPs), DSPE-PEG-PGP and DSPE-PEG (9 mg) at a weight ratio of 1:5 were co-dissolved in deionized water (9.4 mL). Meanwhile, lecithin (6 mg) dispersed in ethanol (0.6 mL) was added into the aqueous solution, followed by heating at 65°C for 1 h to obtain an aqueous phase. After the obtained solution was cooled down to 20-25°C, it was added dropwise into 2 mL of DMF containing 50 mg LCD and 0.2 mg PPV, and then incubated at 20-25°C for 2 h. Subsequently, the organic solvent was removed by centrifugation and repeated washing to collect the final product LAD-PGP NPs. According to similar procedures, DSPE-PEG alone was used to prepare a PEGylated LAD NPs (i.e., LAD-PEG NPs).

**Characterization of Different NPs.** Particle size, size distribution profiles, and ζ-potential values of NPs were detected with a Malvern Zetasizer Nano ZS instrument at 25°C. Transmission electron microscopy (TEM) observation was conducted on a TECNAI-10 microscope (Philips, Netherlands).

**Luminescence Properties of LAD NPs.** Chemiluminescence spectra of LAD NPs (15 mg/mL) at 100 mM ClO^-^ was collected by a fiber optic spectrometer. Concentration-dependent luminescence of NPs at 10 mM H_2_O_2_, H_2_O_2_-responsive luminescence of 10 mg/mL LAD NPs, and time-lapse luminescent signals of LAD NPs (10 mg/mL) at 80 mM H_2_O_2_ were detected by a BPCL ultra weak luminescence analyzer.

Next, the concentration-dependent luminescence of LAD NPs was explored by IVIS spectrum imaging system. After mixing different concentrations of NPs and H_2_O_2_ (the final concentration was 80 mM), luminescence images were collected by a PerkinElmer IVIS Spectrum imaging system and the luminescence intensities were quantified.

Moreover, luminescence intensities of LAD NPs in black 96-well black microplates were quantified by a PerkinElmer IVIS Spectrum imaging system under different conditions. By mixing different concentrations of H_2_O_2_ and LAD NPs (20 mg/mL) solutions, the correlation between the luminescence intensity and the concentration of H_2_O_2_ was observed. To examine the effect of MPO on luminescence of LAD NPs, 100 µL of aqueous solution containing different concentrations of MPO, 100 µL of aqueous solution of NaCl (150 mM), and 100 µL of LAD NPs solution (20 mg/mL) were mixed in a 96-well black microplate. Then 100 µL of 5 mM H_2_O_2_ was added into each well. Immediately, the microplate was imaged (exposure time = 5 min, f/stop = 1, binning = 8, no optical filter). The luminescent signals were also quantified by the IVIS Spectrum instrument.

In a separate experiment, LAD NPs or LCD NPs (10 mg/mL) was mixed with 80 mM H_2_O_2_ in a black 96-well plate. Then luminescence images were acquired with an IVIS Spectrum image system (emission filter, 600-700 nm in 20 nm steps; exposure time, 5 min).

**In Vitro Tissue Penetration Capability of LAD NPs Luminescence.** To investigate tissue penetration capability of LAD NPs luminescence, pork-ham was used to simulate the animal tissues. First, 100 μL of aqueous solution (15 mg/mL) of LAD NPs and LCD NPs was added into a black 96-plate, into which 100 μL of 80 mM H_2_O_2_ was added separately. Immediately, the plate was covered with different layers of the ham piece, the thickness of each piece was about 3 mm. In vitro luminescence images were acquired (exposure time = 5 min, f/stop = 1, binning = 8, no optical filter). The luminescence intensities were analyzed.

**Animals.** BALB/c mice (6-8 weeks) were provided by the Animal Center of the Army Medical University. All animal care and experimental procedures were approved by the Animal Ethical and Experimental Committee of the Army Medical University (Third Military Medical University) (Chongqing, China). Animals were subjected to acclimatization to the laboratory for at least 7 days before experiments.

**Isolation of Mouse Peritoneal Neutrophils.** To induce the production of neutrophils, BALB/c mice were intraperitoneally (i.p.) injected with 3 wt% thioglycolate. After 4 h, peritoneal cells were collected with cold HBSS. Cells were incubated with DMEM containing 10% FBS, penicillin, and streptomycin. After 10 min, non-adherent cells were removed by gentle washing with HBSS. Then peritoneal neutrophils were collected for further studies.

**Cellular Uptake.** Peritoneal neutrophils were planted in 12-well plates (5 × 10^5^ cells per well). After incubation in growth medium for 10 min, the culture medium was changed to 1 mL of fresh medium containing LAD NPs at 20 μg/mL. After incubation for different time periods, cells were rinsed with PBS, fixed with paraformaldehyde (4%), and stained with DAPI. Confocal laser scanning microscopy (CLSM) observation was conducted by a confocal microscope (Leica, Heidelberg, Germany). Through similar procedures, cellular uptake of LAD-PEG NPs or LAD-PEG-PGP NPs at 20 μg/mL was examined by CLSM.

To quantify internalized LAD NPs, peritoneal neutrophils were seeded in a 12-well plate (1 × 10^6^ cells per well) and incubated in culture medium for 10 min. Then the medium was switched to 1 mL of medium containing LAD NPs at 20 μg/mL. After incubation for predetermined time periods, cells were digested and fluorescence intensities were quantified by flow cytometry (BD FACS Calibur). Cellular uptake of LAD-PEG NPs or LAD-PEG-PGP NPs at 20 μg/mL was examined through similar same procedures.

To address the specific pathways dominating cellular uptake of LAD NPs in neutrophils, cells were separately pretreated with different inhibitors (including amiloride, nocodazole, chlorpromazine, genistein, and sodium azide), and then co-incubated with LAD NPs (at 5 μg/mL) for 1 h, followed by quantification via flow cytometry.

**Stability of LAD NPs in Neutrophil Lysates**

Briefly, mouse peritoneal neutrophils (5 × 10^6^ or 1 × 10^7^) were mixed with 1.5 mL of PBS and stimulated with PMA, followed by centrifugation at 18000*g* for 20 min to destroy the cell structure. Then 1 mL of the collected supernatant was incubated with 3 mg LAD NPs. After different time periods, the mean diameter of LAD NPs was determined by DLS.

**Luminescence Imaging of Activated Neutrophils by LAD NPs.** Peritoneal neutrophils (5 × 10^5^ cells per well) were planted in 12-well plates, which were stimulated with or without 100 ng/mL PMA for 1 h. Subsequently, cells were treated with 3 mg/mL LAD NPs. The time-dependent changes in luminescence signals of LAD NPs in different groups were examined at various time points by an IVIS imaging system (exposure time = 5 min, f/stop = 1, binning = 8, no optical filter). In another study, activated neutrophils were incubated with LAD NPs of different concentrations for 1 h, and the luminescent signals were quantified. The concentration-dependent luminescence of neutrophil counts was examined by similar procedures.

In a separate study, luminescence performance of different NPs including LAD NPs, LAD-PEG NPs, and LAD-PGP NPs were compared. To this end, peritoneal neutrophils (5 × 10^5^ cells per well) were planted in 12-well plates and PMA (100 ng/mL) was added into each well to stimulate the cells for 1 h. Subsequently, cells were incubated separately with 3 mg/mL different NPs, followed by luminescence imaging (exposure time = 5 min, f/stop = 1, binning = 8, no optical filter).

**Establishment of a Lung Metastatic Tumor Model in Mice.** To establish a mouse metastatic tumor model in mice, 4T1-GFP breast cancer cells (1 × 10^5^ cells) suspended in 100 μL of PBS were injected intravenously into the tail vein of each mouse. At different time points after injection, mice were sacrificed for different studies.

**Collection of Bronchoalveolar Lavage Fluid (BALF).** At specific time points after different treatments, mice were euthanized. The lung was exposed by removing the diaphragm, and blunt dissection was carried out to expose the principal bronchus. An oblique incision was made at the trachea, and a small gavage needle was inserted, the gastric bypass and the tracheal junction were then ligated with surgical sutures. Then 1 mL of PBS was slowly injected into the lung of mice, and this procedure was repeated three times. After staying for 2-3 seconds, bronchoalveolar lavage was slowly extracted for different analyses.

**Analysis of the Levels of Neutrophils, MPO, and H_2_O_2_ in BALF of Mice with Inoculated 4T1-GFP Cells.** At defined time points post i.v. injection of 4T1-GFP tumor cells in mice, BALF was collected. Then 1 mL of BALF was taken and centrifuged at 450g, followed by 2 min of incubation with ACK lysing buffer (Beyotime) to exhaust erythrocytes. Subsequently, cells were labeled with APC-conjugated rat anti-mouse CD11b and PE-conjugated rat anti-mouse Ly6G antibodies for analysis by flow cytometry. In addition, 1 mL of BALF was centrifuged at 21000g, the MPO level in the collected supernatant was quantified by ELISA. The H_2_O_2_ concentration in the supernatant was quantified by the corresponding kit. In a separate experiment, lung tissues from mice at different time points after inoculation of 4T1-GPF cells were excised and digested for flow cytometric analysis.

**In Vivo Targeting Capability of Different NPs in Mice with Inoculated 4T1-GFP Cells.** Mice were inoculated with 4T1-GFP cells by i.v. injection. After 3 weeks, different NPs were separately administered by i.v. injection at 3 mg in each mouse. Meanwhile, PBS was injected in the control mice. Mice were euthanized at 10-15 min post injection for in vivo luminescence imaging, and then whole lungs were isolated for ex vivo fluorescence imaging. Both ex vivo fluorescence imaging (exposure time = 10 s, f/stop = 1, binning = 8, no optical filter) and in vivo luminescence imaging (exposure time = 5 min, f/stop = 1, binning = 8, no optical filter) were conducted by a PerkinElmer IVIS Spectrum System.

In a separate study, 4T1-GFP cell-inoculated mice at week 3 were treated with LAD-PGP NPs by i.v. administration at 3 mg in each mouse. Meanwhile, healthy mice injected with LAD-PGP NPs served as the control. Mice were euthanized at 12 h post injection, and whole lungs were isolated and analyzed by CLSM.

**Luminescence Imaging of Lung Metastasis in Mice.** At weeks 2, 3, 4, and 5 after inoculation of 4T1 cells in mice, each animal was administered with 3 mg LAD NPs by i.v. injection. After 5 min, mice were anaesthetized by isoflurane, and in vivo luminescence images were acquired (exposure time = 5 min, f/stop = 1, binning = 8, no optical filter).

In another experiment, 4T1-GFP cell-inoculated mice were separately imaged in the luminescence or fluorescence mode by an in vivo Xtreme II imaging system (Bruker, USA). The luminescence observation group first received intraperitoneal injection of pentobarbital for anesthesia, and then received i.v. injection of 3 mg LAD-PGP NPs in each mouse, followed by in vivo luminescence imaging, with an exposure time of 5 min. Lung tissues were then immediately isolated for ex vivo imaging under the same conditions. For fluorescence observation, mice were also anesthetized first, and then in vivo images were acquired in the fluorescence mode with an exposure time of 10 s. Also, isolated lung tissues were subjected to ex vivo imaging in the fluorescence mode with the same exposure time.

**Comparison of Different Clinical Imaging Modalities.** At weeks 2, 3, 4, and 5 after 4T1-GFP cells were inoculated in mice by i.v. injection, mice were imaged by PET and CT with the Mediso nanoScan PET/CT equipment. Mice were placed in separate cages one day before operation without water and food to reduce metabolism. For PET imaging, each mouse was i.v. injected with 200-300 μCi ^18^F-FDG, and then placed on a warm mat to reduce the metabolic uptake of brown fat on the back. After 40 min, mice were anesthetized with isoflurane and PET images were collected for 20 min. Subsequently, CT images were also collected for 20 min.

**Comparison of Blood Neutrophil Levels in Mouse Models of Lung Metastasis and Lung Infection.** An aqueous solution of cyclophosphamide (CTX) (Lot. MKCG546; Sigma-Aldrich, USA) was freshly prepared and sterilized by passing through a 0.2-µm syringe filter. Mice were injected (i.v.) with 150 mg/kg and 100 mg/kg CTX at day 0 and day 3. After intraperitoneal anesthesia using 1% pentobarbital sodium solution (50 mg/kg), mice were fixed in a supine position for administration of K. pneumoniae to the lung at day 4. To this end, the trachea was exposed, 100 µL of the K. pneumoniae suspension at 3 × 10^8^ colony forming units (CFU) per mL was slowly injected into the mouse trachea. Mice in the control group were intraperitoneally injected with saline. After 24 h infection, mice were euthanized to collect blood samples for quantification of neutrophil counts, which were compared with those of mice with 4T1 lung metastasis.

**Acute Toxicity Tests.** BALB/c mice were randomly divided into 3 groups (n = 6). Mice in the two LAD NPs groups were i.v. administered with LAD NPs at 500 or 1000 mg/kg, while mice in the control group were i.v. injected with saline. After administration, the body weight of mice and their general behaviors were monitored for any signs of diseases each day. At day 15, mice were euthanized. Blood samples were collected for hematological analysis and quantification of biochemical markers relevant to liver/kidney functions. Major organs including heart, liver, spleen, lung, and kidney were harvested and weighed. Histological sections were prepared and stained with hematoxylin-eosin (H&E).

**Statistical Analysis.** All quantitative data are presented as means ± standard deviation (SD). Statistical analysis was conducted with the software of SPSS23 using a one-way ANOVA test with multiple comparisons for experiments containing more than two groups. The one-way ANOVA with a two-tailed, unpaired t-test was performed for two-group experiments. P < 0.05 is considered statistically significant.

**References**

[1] J. M. Shi, S. Y. Zheng, *Macromolecules* **2001**, *34*, 6571-6576.


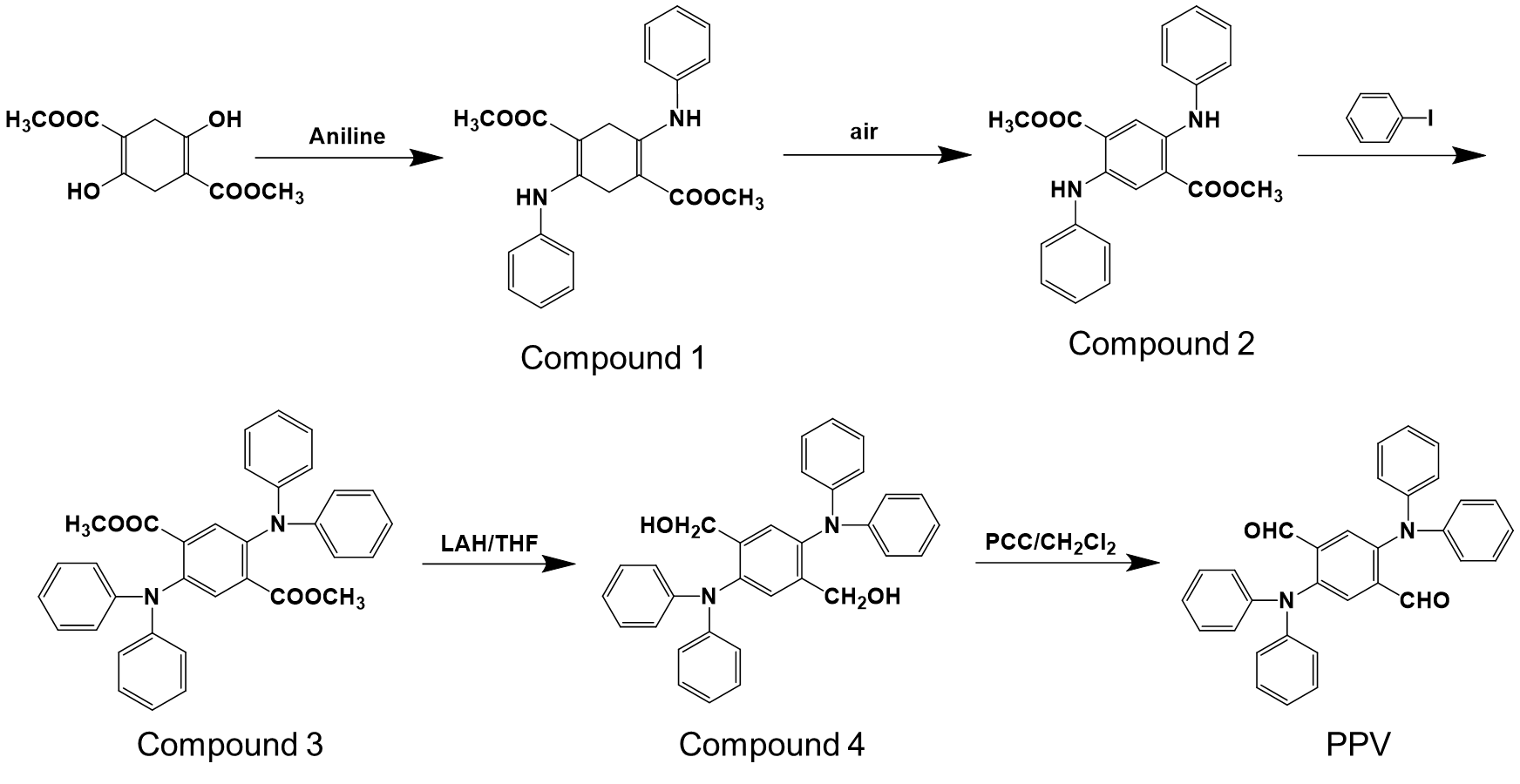


**Scheme S1.** Synthesis of PPV.


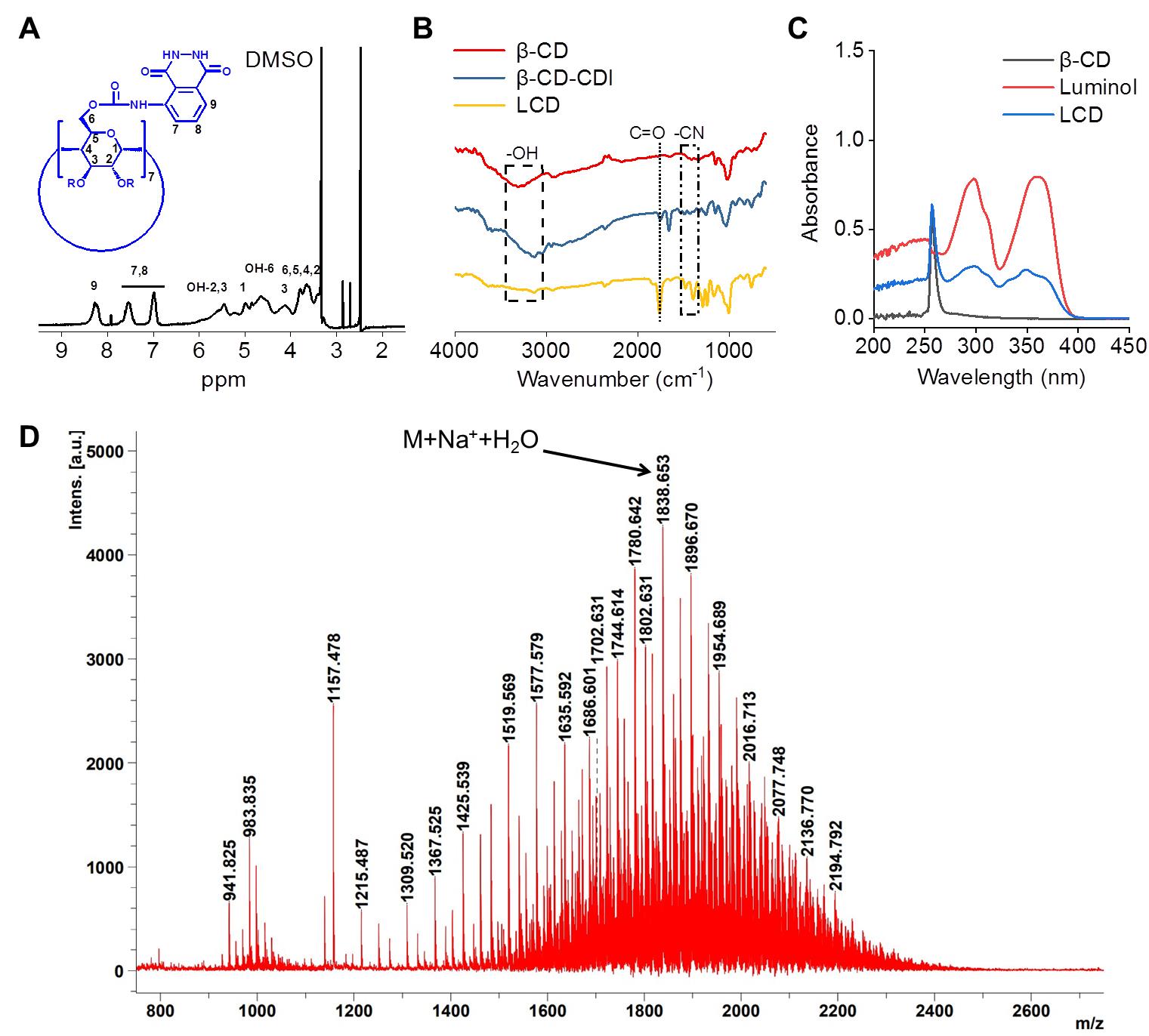


**Fig. S1** Structural characterization of a luminescent material LCD. (A) ^1^H NMR spectrum of LCD in DMSO-d_6_. (B) FT-IR spectra of different materials including β-CD, CDI-activated β-CD (CDI-CD), and luminol-conjugated β-CD (LCD). (C) UV-Vis absorbance spectra of different materials in DMSO. (D) The MALDI-TOF mass spectrum of LCD.


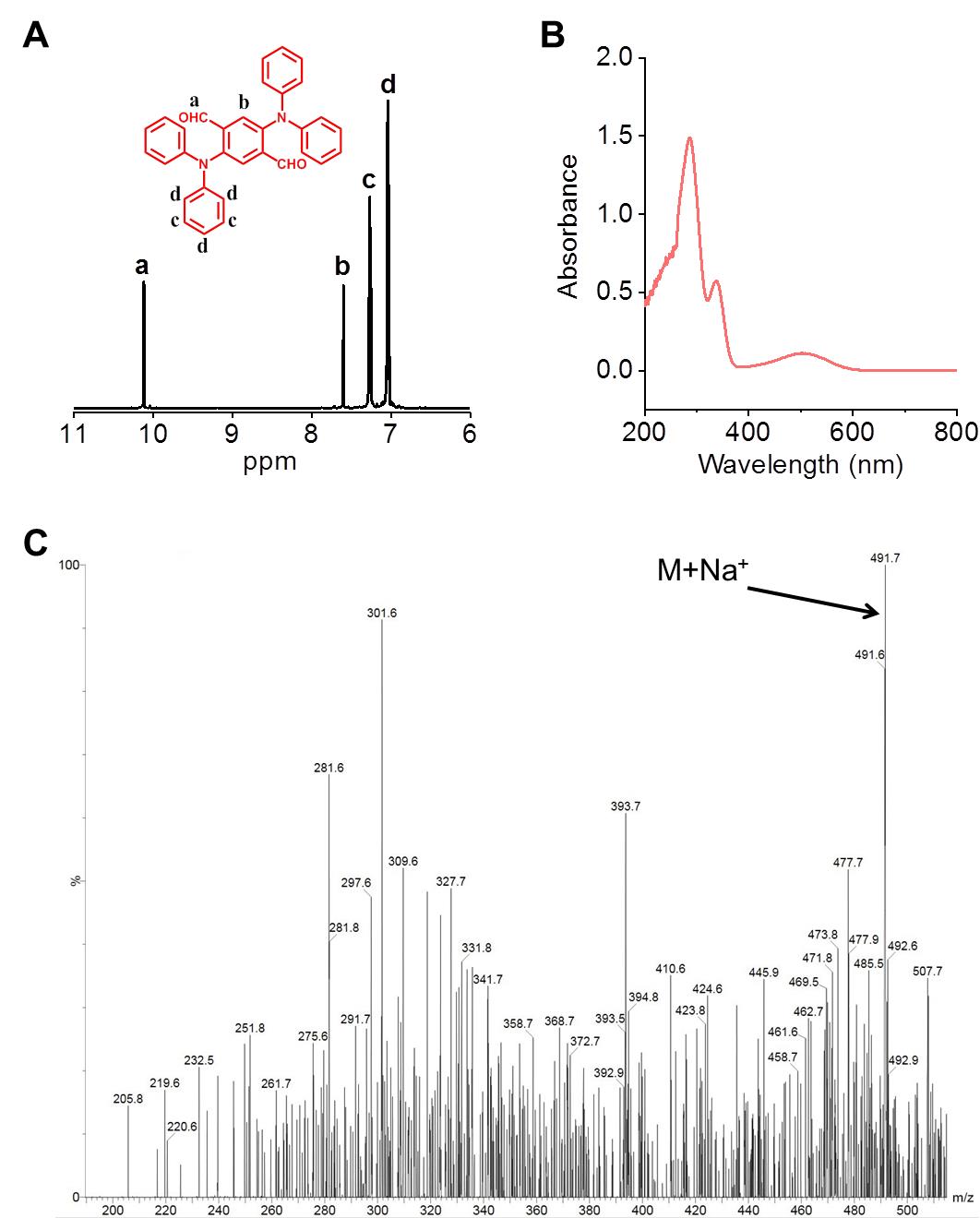


**Fig. S2** Characterization of PPV. (A) ^1^H NMR spectrum of PPV in CDCl_3_. (B) UV-Vis spectrum of PPV in DMSO. (C) The LC-MS spectrum of PPV.


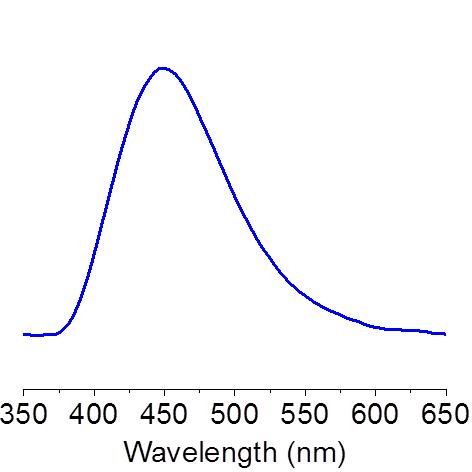


**Fig. S3** The luminescence spectrum of luminol in the presence of hypochlorite.

**
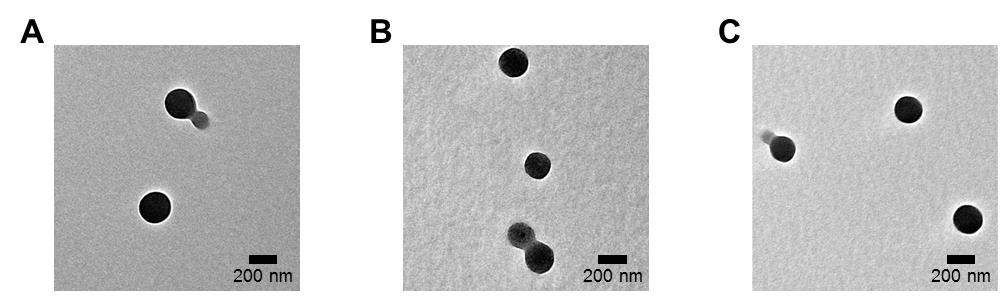
**

**Fig. S4** TEM images of LAD NPs containing various contents of PPV. (A-C) The PPV contents were 0.2% (A), 0.4% (B), and 0.8% (C).


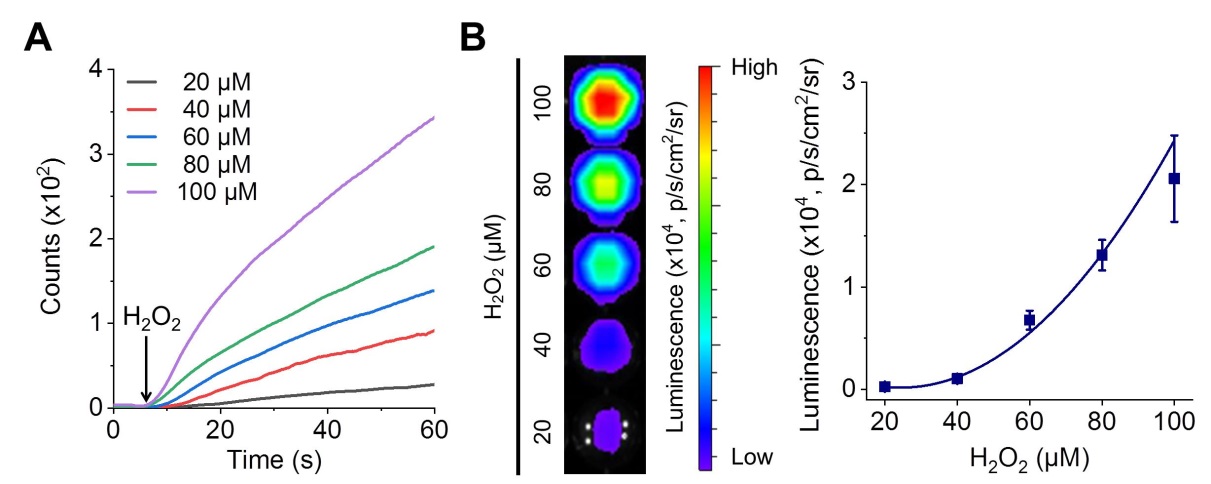


**Fig. S5** Luminescence profiles of LAD NPs at pathophysiological concentrations of H_2_O_2_. (A) Time-lapse luminescence curves of LAD NPs at 20 mg/mL upon incubation with various levels of H_2_O_2_. (B) Effects of H_2_O_2_ levels on luminescent signals of LAD NPs at 20 mg/mL. Data in (B) are expressed as mean ± SD (n = 3).


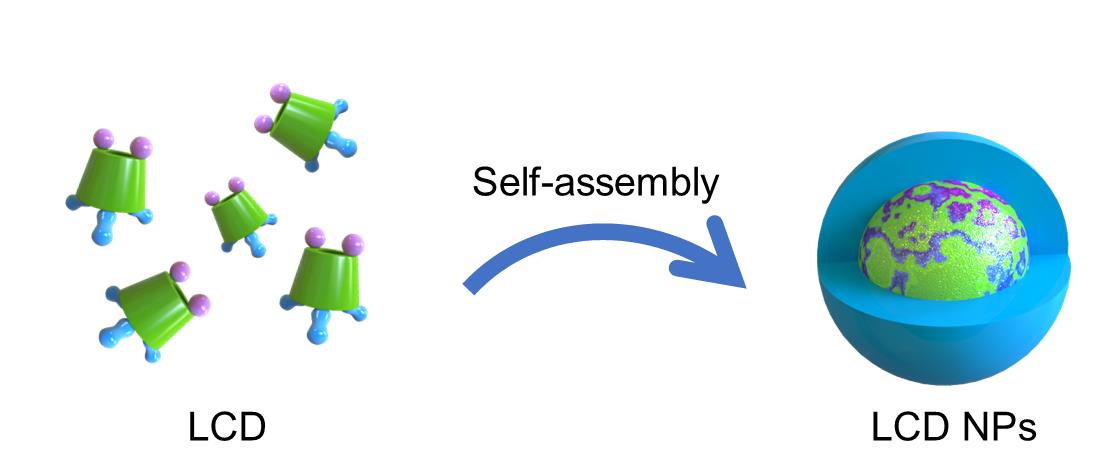


**Fig.** **S6** A sketch showing preparation of LCD nanoparticles (LCD NPs) by nanoprecipitation.


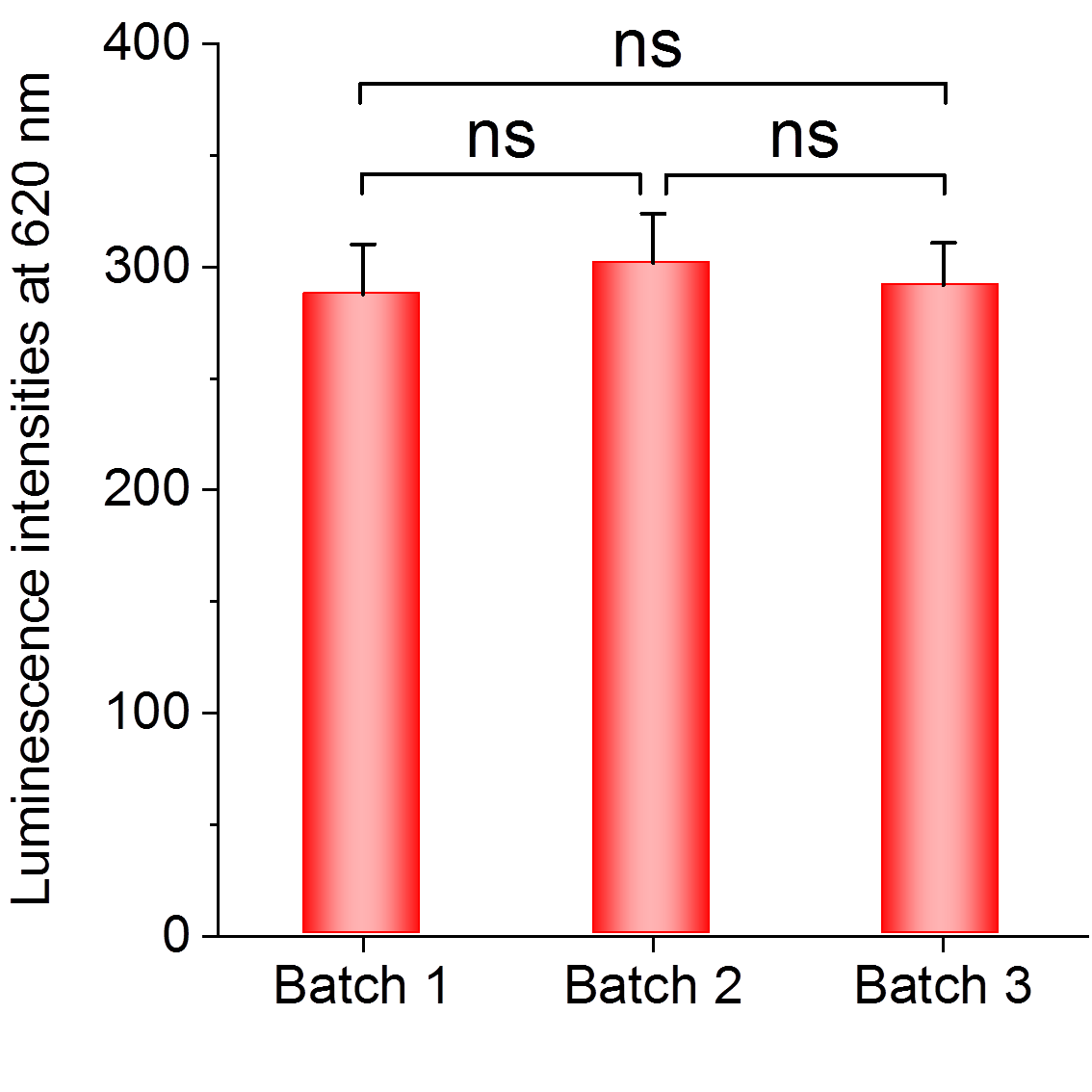


**Fig.** **S7** Comparison of luminescence intensities of LAD NPs based on three different batches in the presence of ClO^-^. The concentration of LAD NPs was 30 mg/mL, while the level of ClO^-^ was 100 mM. In all cases, the integration time was 10 ms. Data are expressed as mean ± SD (n = 3). ns, no significance.


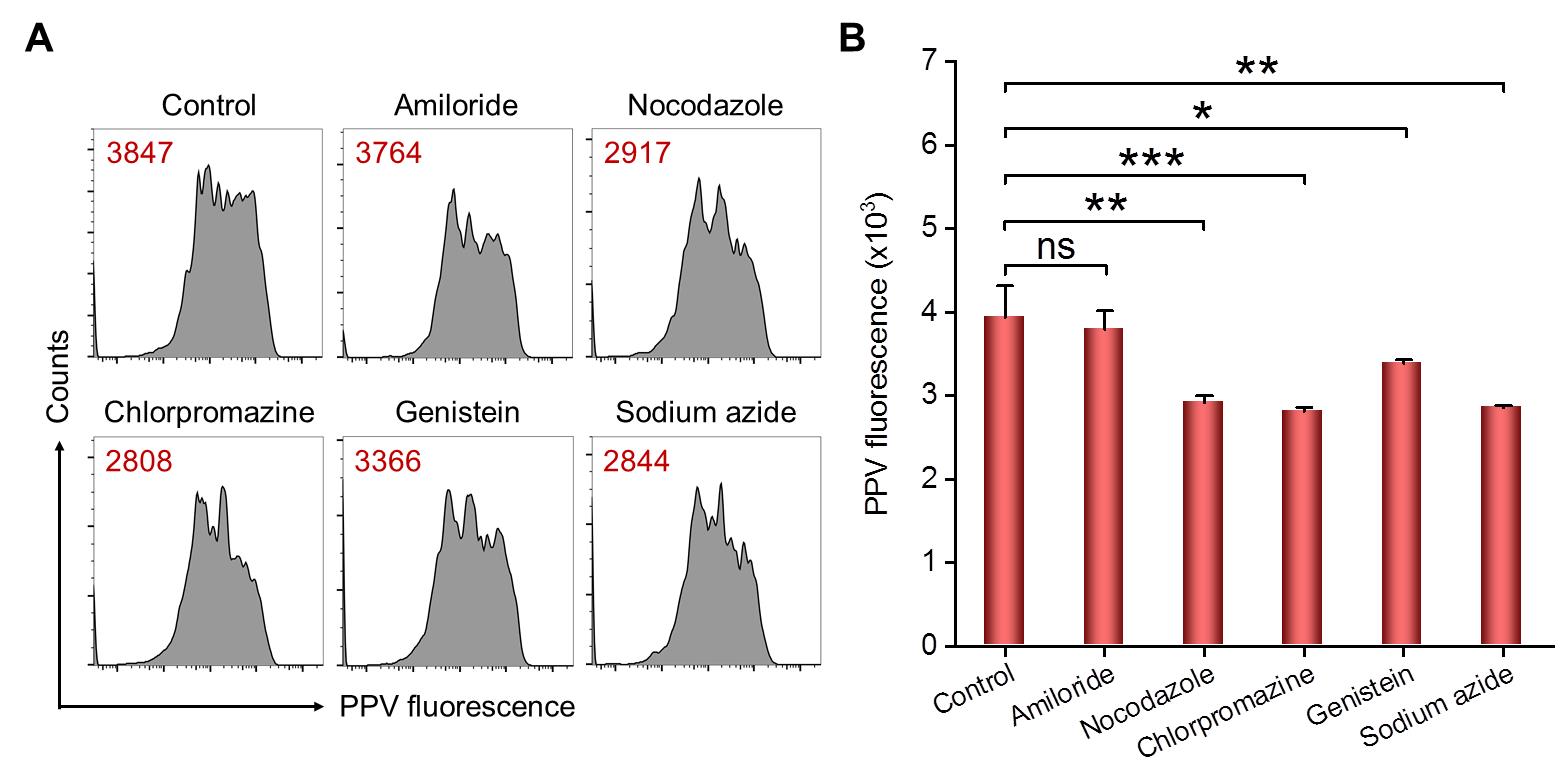


**Fig. S8** The effects of different inhibitors on cellular uptake of LAD NPs in neutrophils. (A-B) Flow cytometric profiles (A) and quantitative results (B) indicating intracellular fluorescence intensities of LAD NPs after neutrophils were pretreated with different inhibitors. In all cases, the dose of LAD NPs was 5 μg/mL, while the incubation time was 1 h. Data in (B) are expressed as mean ± SD (n = 4). **P* < 0.05, ***P* < 0.01, ****P* < 0.001; ns, no significance.


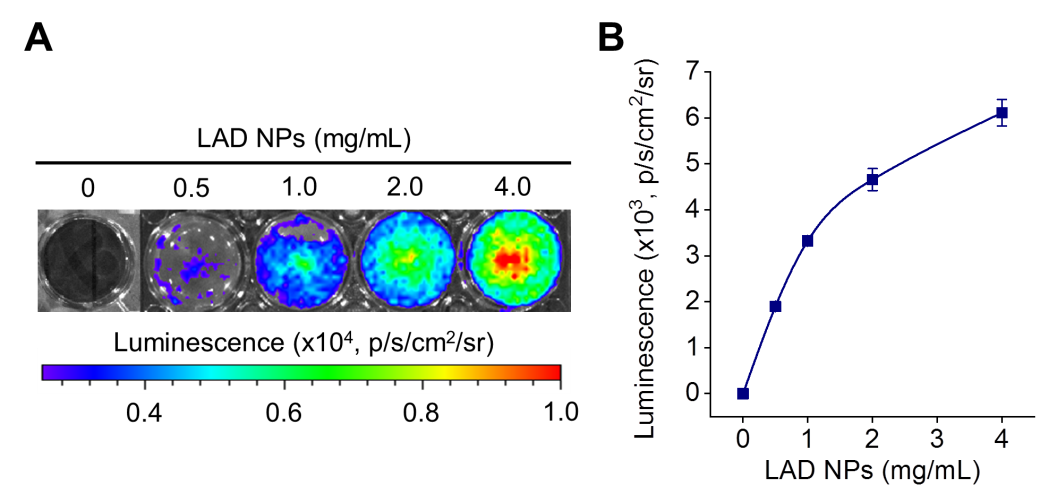


**Fig. S9** Dose-dependent luminescence of LAD NPs in peritoneal neutrophils. (A-B) Representative luminescence images (A) and quantitative data (B) showing luminescence intensities after peritoneal neutrophils (5 × 10^5^ cells per well) were incubated with different doses of LAD NPs for 5 min. Data are expressed as mean ± SD (n = 3).


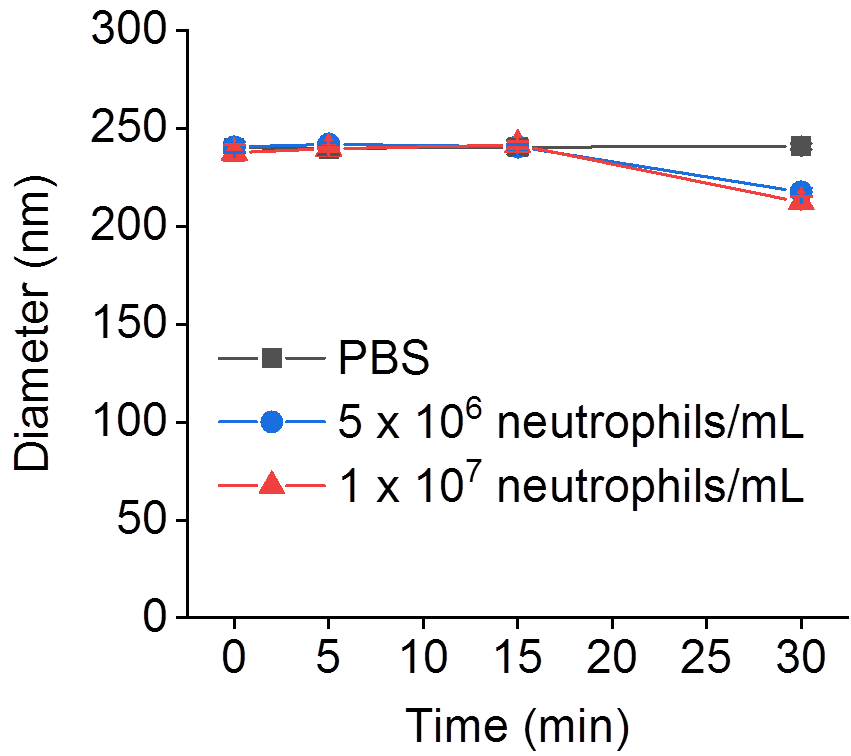


**Fig. S10** Changes in the mean diameter of LAD NPs after incubation with PBS or neutrophil lysates. The cell lysates were prepared at 5 × 10^6^ or 1 × 10^7^ neutrophils per mL PBS. Data are expressed as mean ± SD (n = 3).


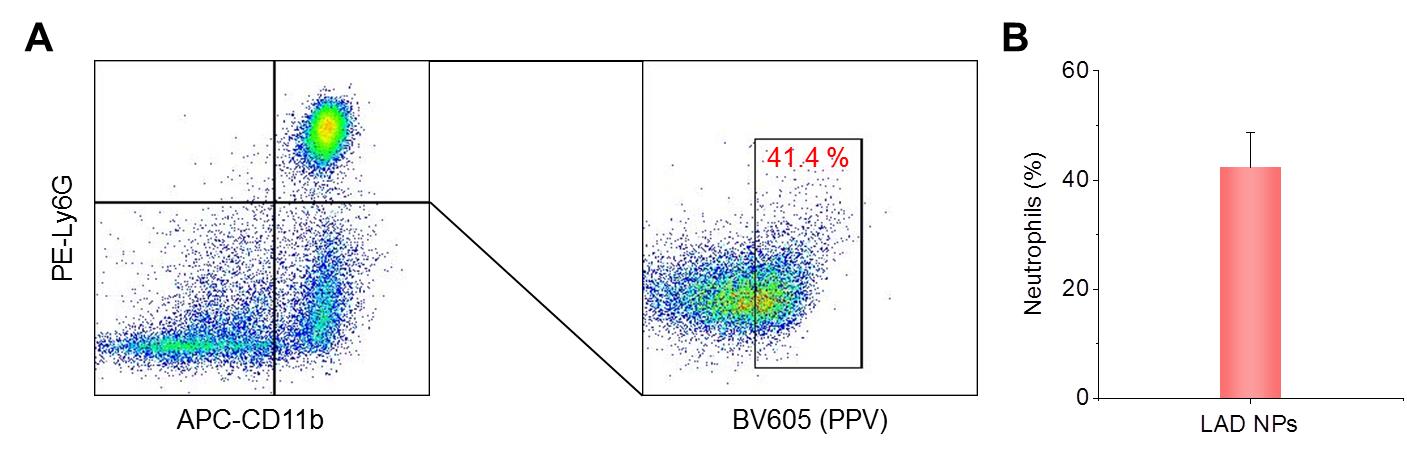


**Fig. S11** Flow cytometric analysis of the distribution of LAD NPs in neutrophils in the lung tissue of mice at week 3 after i.v. inoculation of 4T1-GFP tumor cells. Lung tissues were isolated at 12 h after i.v. injection of LAD NPs. (A-B) Flow cytometric profiles (A) and quantitative analysis (B) of LAD NPs-positive neutrophils. Data in (B) are expressed as mean ± SD (n = 4).


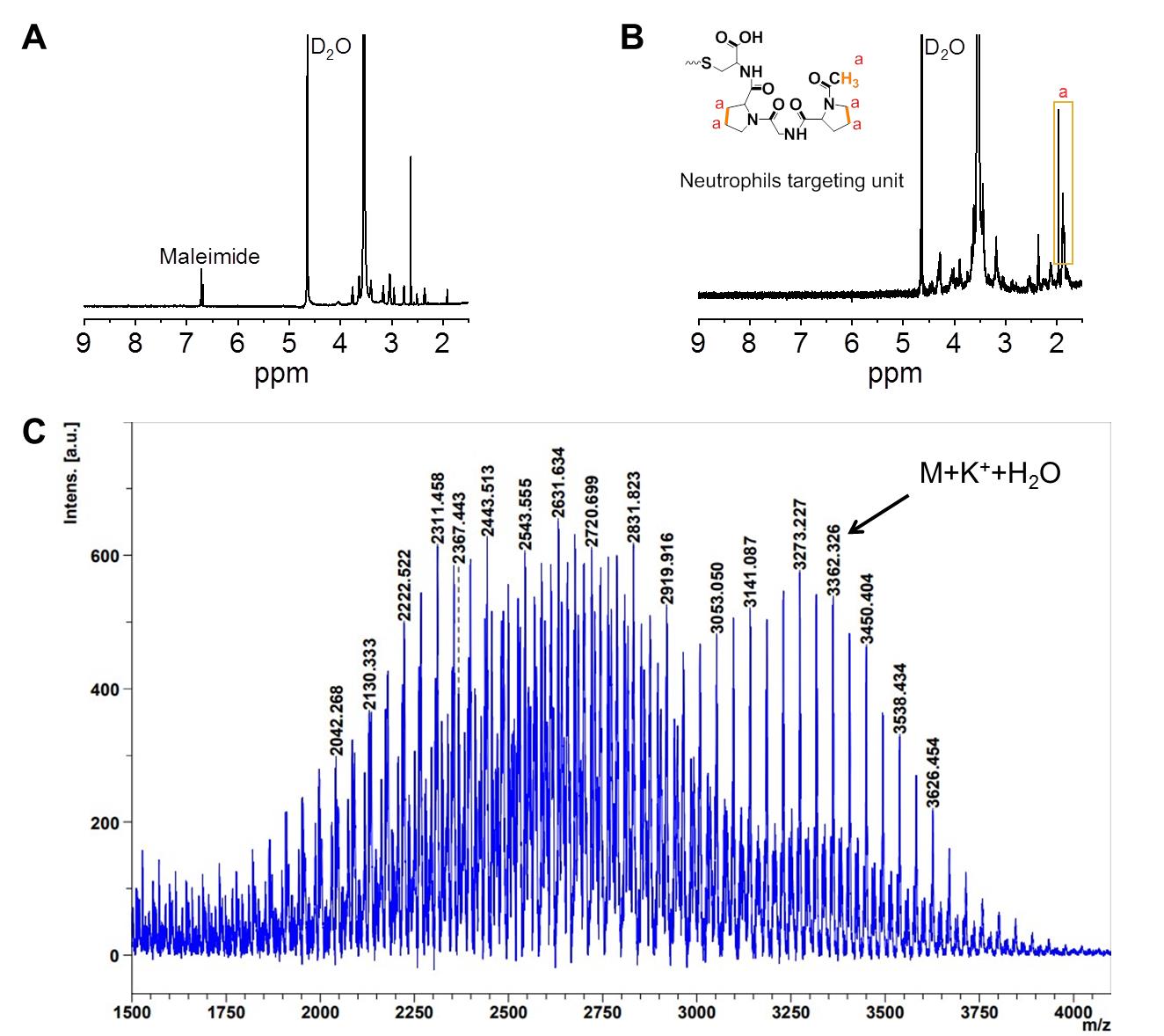


**Fig. S12** Spectroscopy characterization of PGP-conjugated DSPE-PEG (DSPE-PEG-PGP). (A-B) ^1^H NMR spectra of DSPE-PEG-Maleimide (A) and PGP-conjugated DSPE-PEG (DSPE-PEG-PGP) (B) in D_2_O. (C) The MALDI-TOF mass spectrum of DSPE-PEG-PGP.


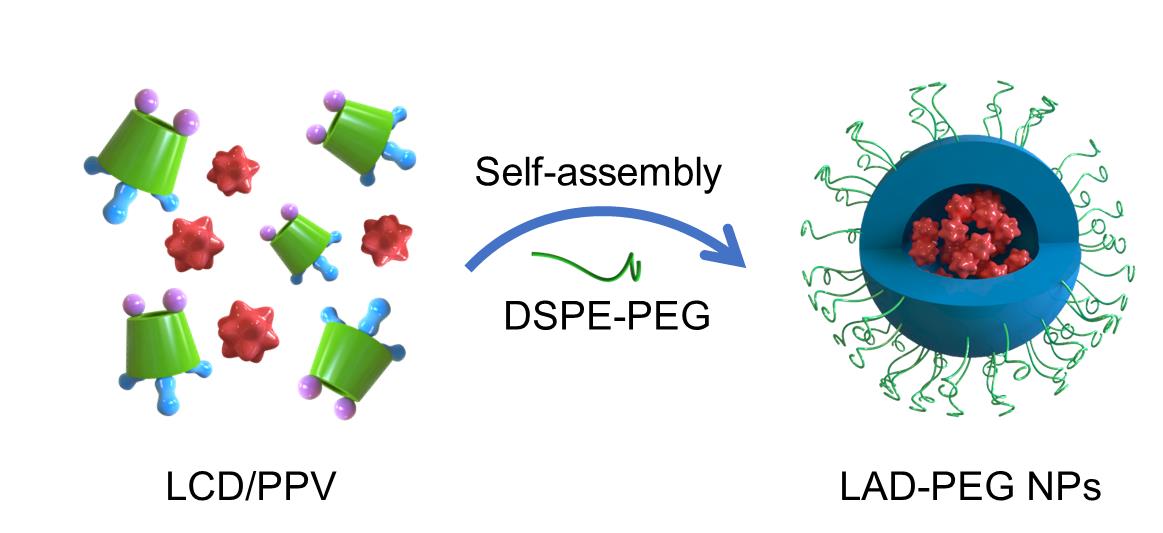


**Fig. S13** Schematic illustration of preparation of PPV-loaded PEGylated LCD NPs (LAD-PEG NPs) by a nanoprecipitation/self-assembly method.


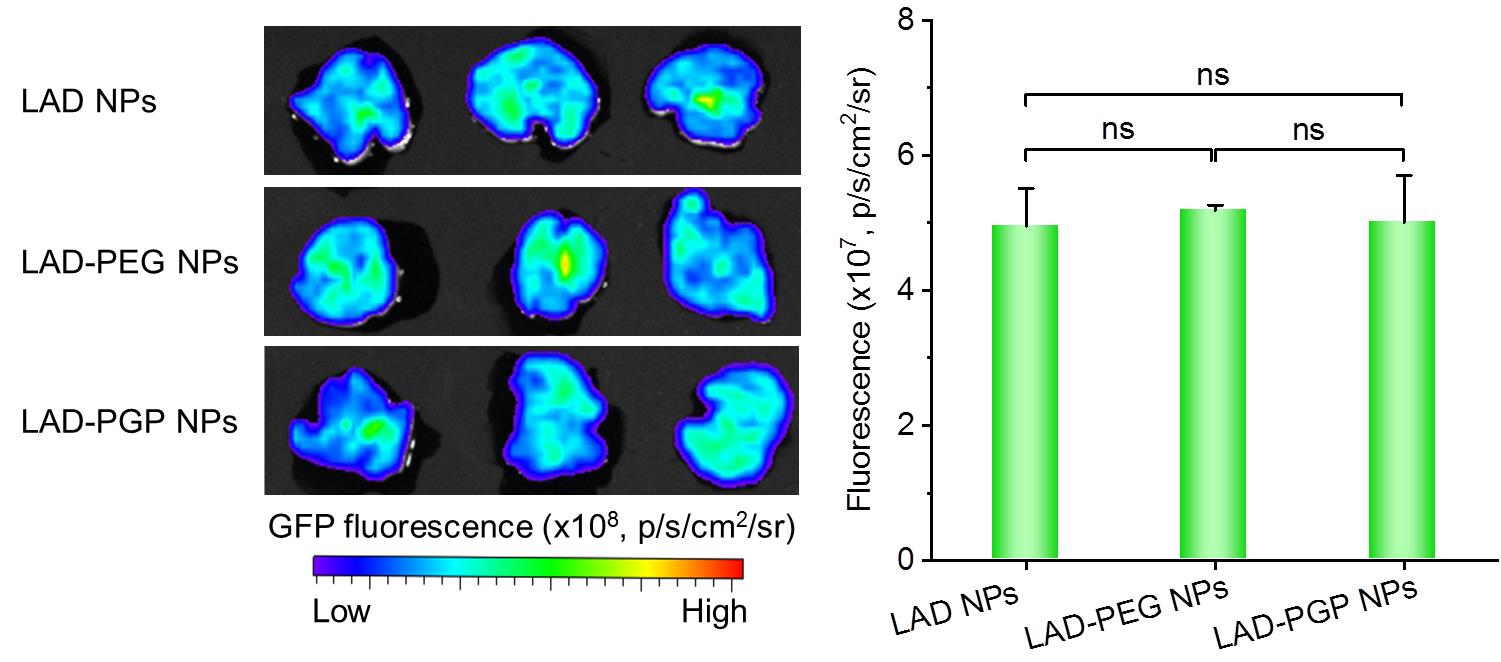


**Fig. S14** Ex vivo imaging of GFP fluorescence intensities in lung tissues of mice at week 3 after i.v. inoculation of 4T1-GFP tumor cells. At 10 min after i.v. injection of 3 mg different NPs in each mouse, lung tissues were isolated for ex vivo imaging via the GFP channel. Data are expressed as mean ± SD (n = 3). ns, no significance.


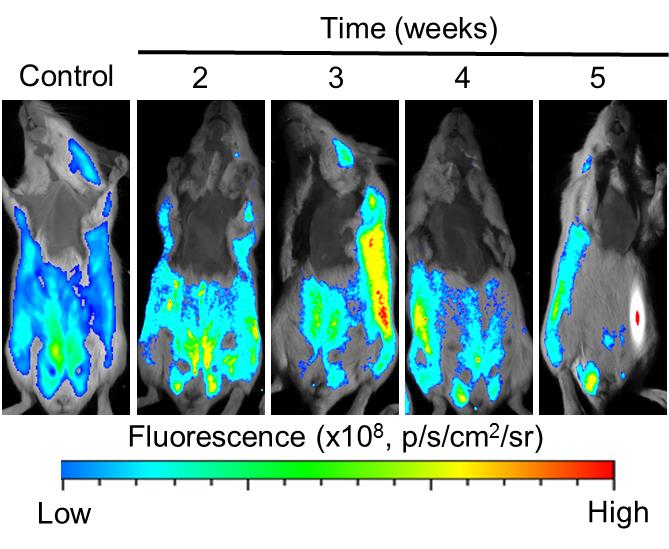


**Fig. S15** In vivo fluorescence images of mice inoculated with 4T1-GFP cells by i.v. injection. At different time points after i.v. inoculation of 4T1-GFP cells in mice, in vivo fluorescence imaging was performed with excitation at 395 nm and exposure time of 10 s. Mice without inoculation of 4T1-GFP cells served as the control.


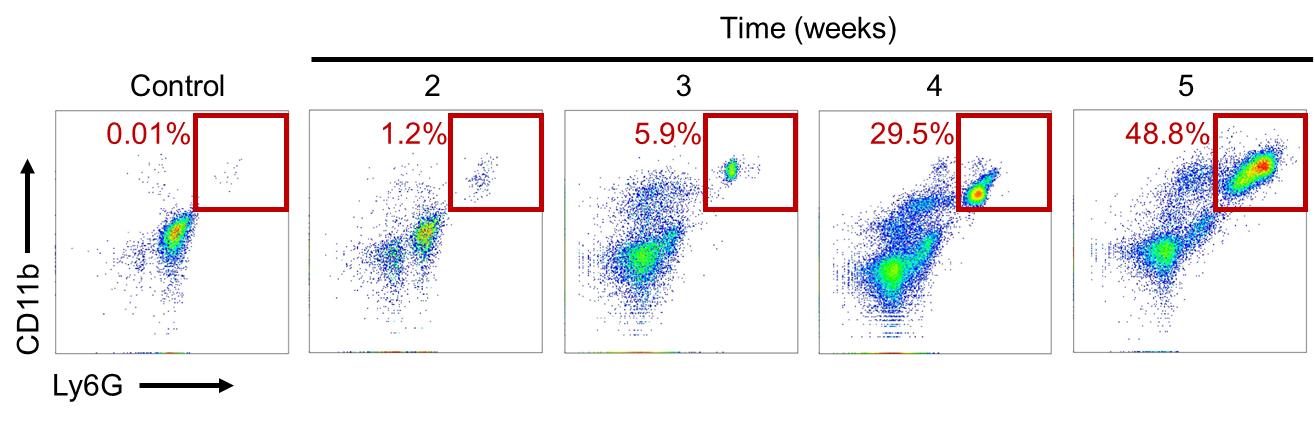


**Fig. S16** Flow cytometric profiles showing neutrophil counts in bronchoalveolar lavage fluid (BALF) from mice with or without inoculation of 4T1 cells. At different time points after i.v. inoculation of 4T1 cells in mice, BALF was collected for analysis. Mice without inoculation of 4T1-GFP cells served as the control.


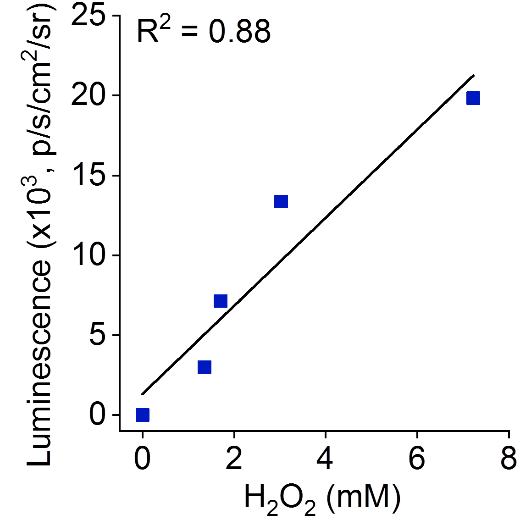


**Fig. S17** Analysis of correlation between the luminescence intensity and the H_2_O_2_ concentration in BALF.


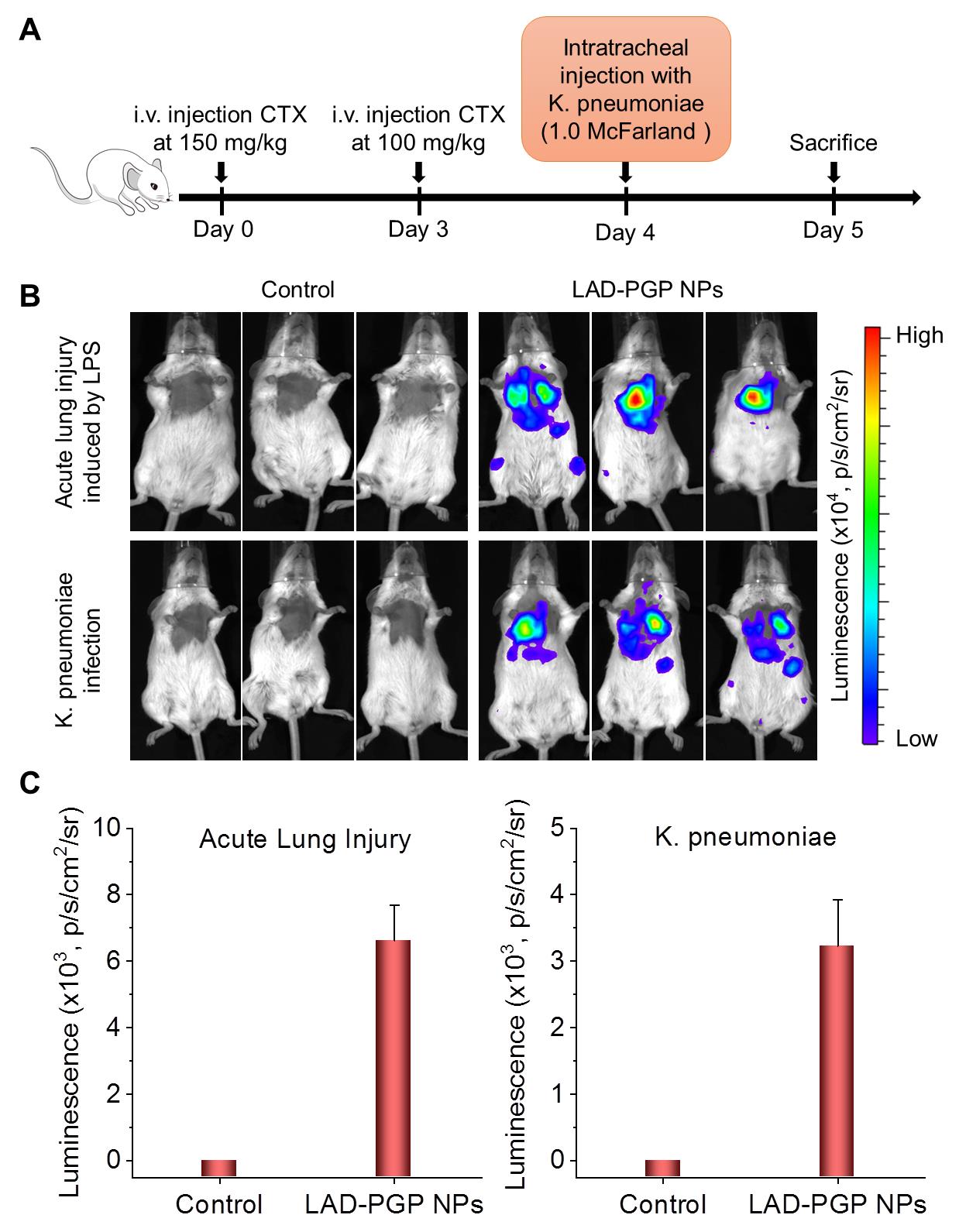


**Fig.** **S18** Luminescence imaging of acute inflammation in the lungs with LAD-PGP NPs. (A) Treatment regimens for the establishment of a mouse model of Klebsiella pneumoniae (K. pneumoniae) lung infection. CTX, cyclophosphamide. (B) In vivo fluorescence imaging of LPS-induced acute lung injury and K. pneumoniae infection in mice after i.v. injection 3 mg LAD-PGP NPs in each animal. Mice were treated with saline in the control group. (C) Quantitative analysis of luminescence intensities. Data in (C) are expressed as mean ± SD (n = 3).


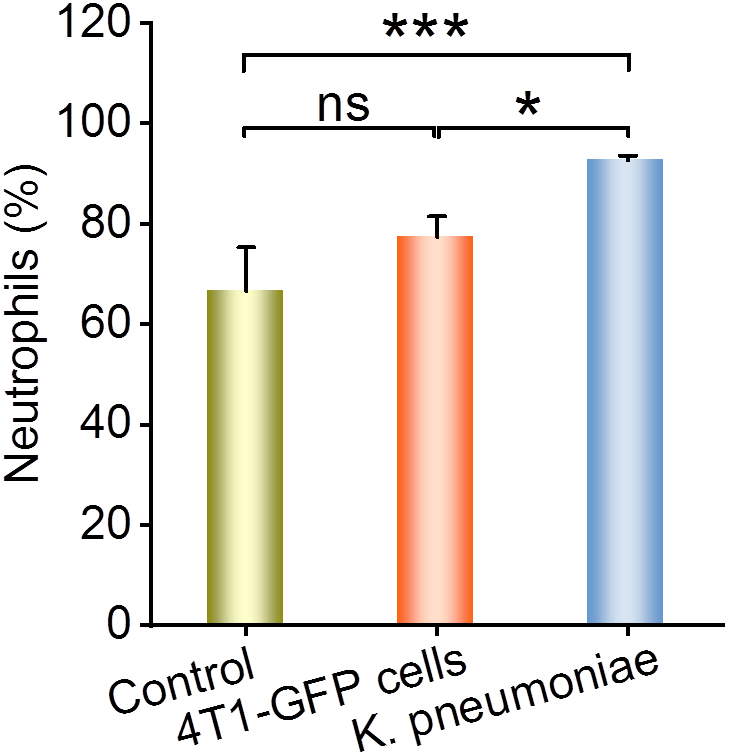


**Fig. S19** Comparison of peripheral blood neutrophils in mice with lung metastasis or lung infection. For the 4T1-GFP cells group, mice were inoculated with 4T1-GFP cells by i.v. injection, followed by blood sampling at week 3. Mice with lung infection with K. pneumoniae were established following the procedures shown in Fig. S18A. Healthy mice were treated with saline in the control group. Data are expressed as mean ± SD (n = 5). **P* < 0.05, ****P* < 0.001; ns, no significance.


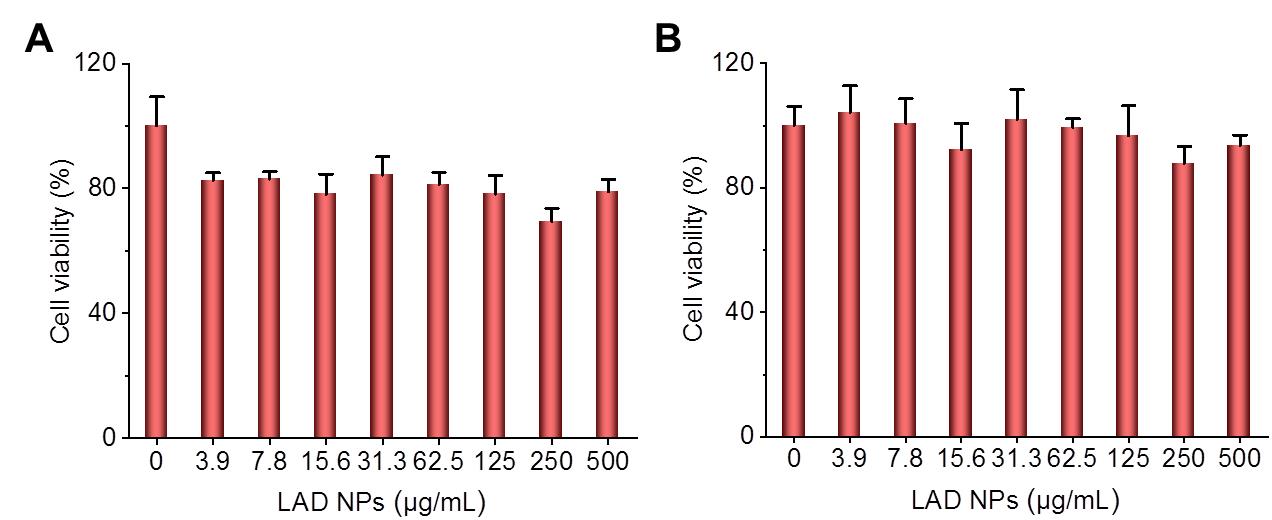


**Fig.** **S20** Cytotoxicity evaluation of LAD NPs. (A-B) Cell viability of 4T1 cells (A) and neutrophils (B) after incubation with various doses of LAD NPs for 12 h. Data are expressed as mean ± SD (n = 6).

**
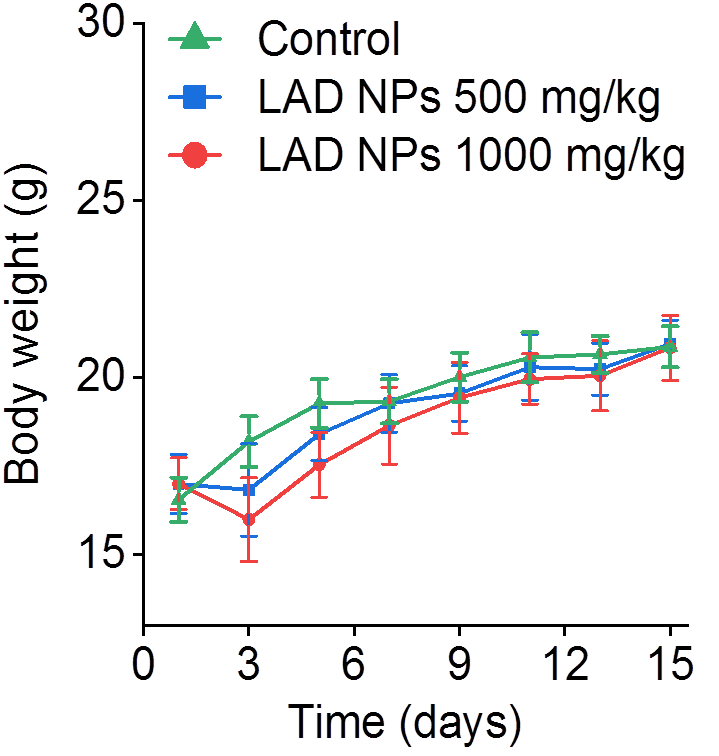
**

**Fig. S21** Changes in mouse body weight after a single *i.v.* administration of LAD NPs at 500 or 1000 mg/kg. Data are expressed as mean ± SD (n = 6).


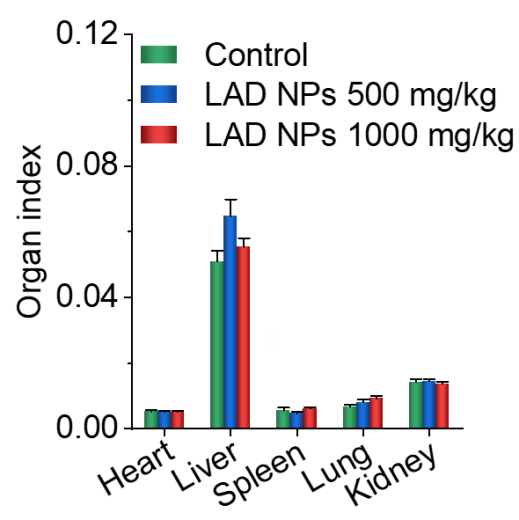


**Fig.** **S22** The organ index of typical major organs at day 15 after treatment with LAD NPs. Data are expressed as mean ± SD (n = 6).

**
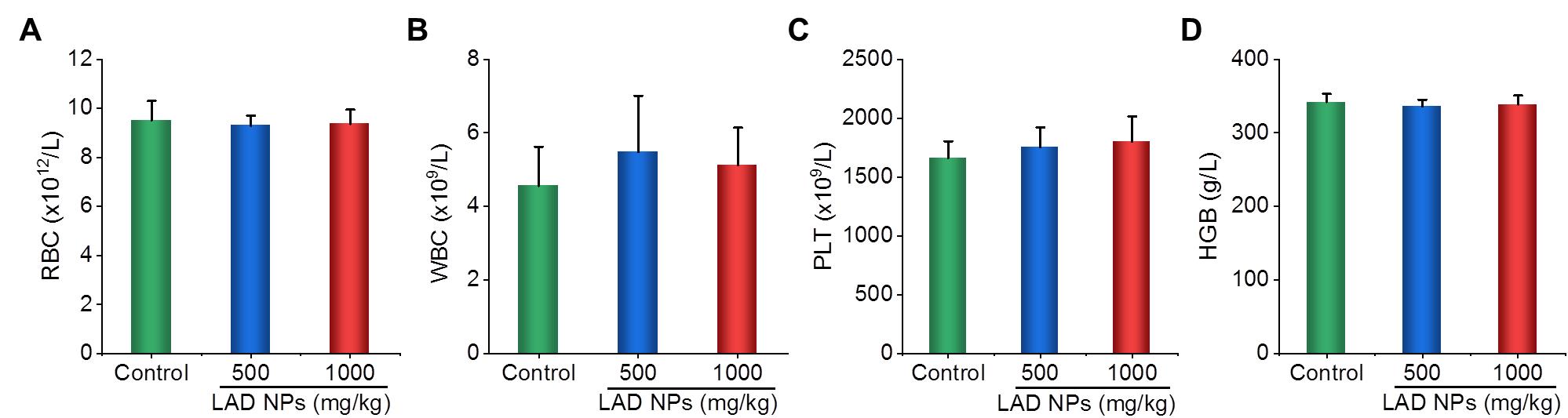
**

**Fig.** **S23** The blood levels of WBC, RBC, PLT, and HGB at day 15 after treatment with various doses of LAD NPs. WBC, white blood cells; RBC, red blood cells; PLT, platelets; HGB, hemoglobin. Data are expressed as mean ± SD (n = 6).


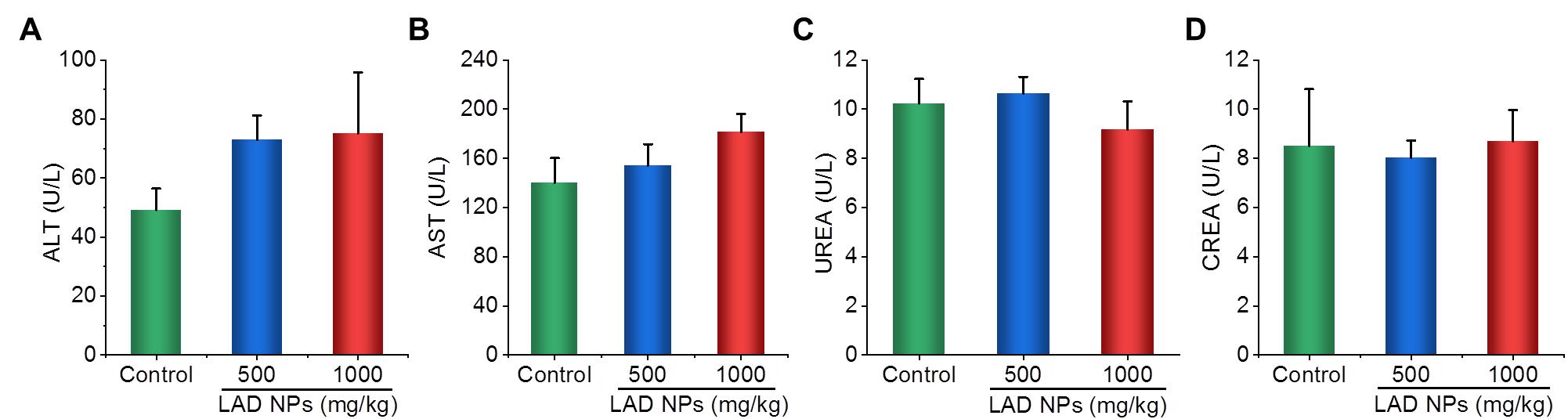


**Fig.** **S24** Serum levels of ALT, AST, UREA, and CREA at day 15 after treatment with various doses of LAD NPs. ALT, alanine aminotransferase; AST, aspartate aminotransferase; UREA, blood urea; CREA, creatinine. Data are expressed as mean ± SD (n = 6).


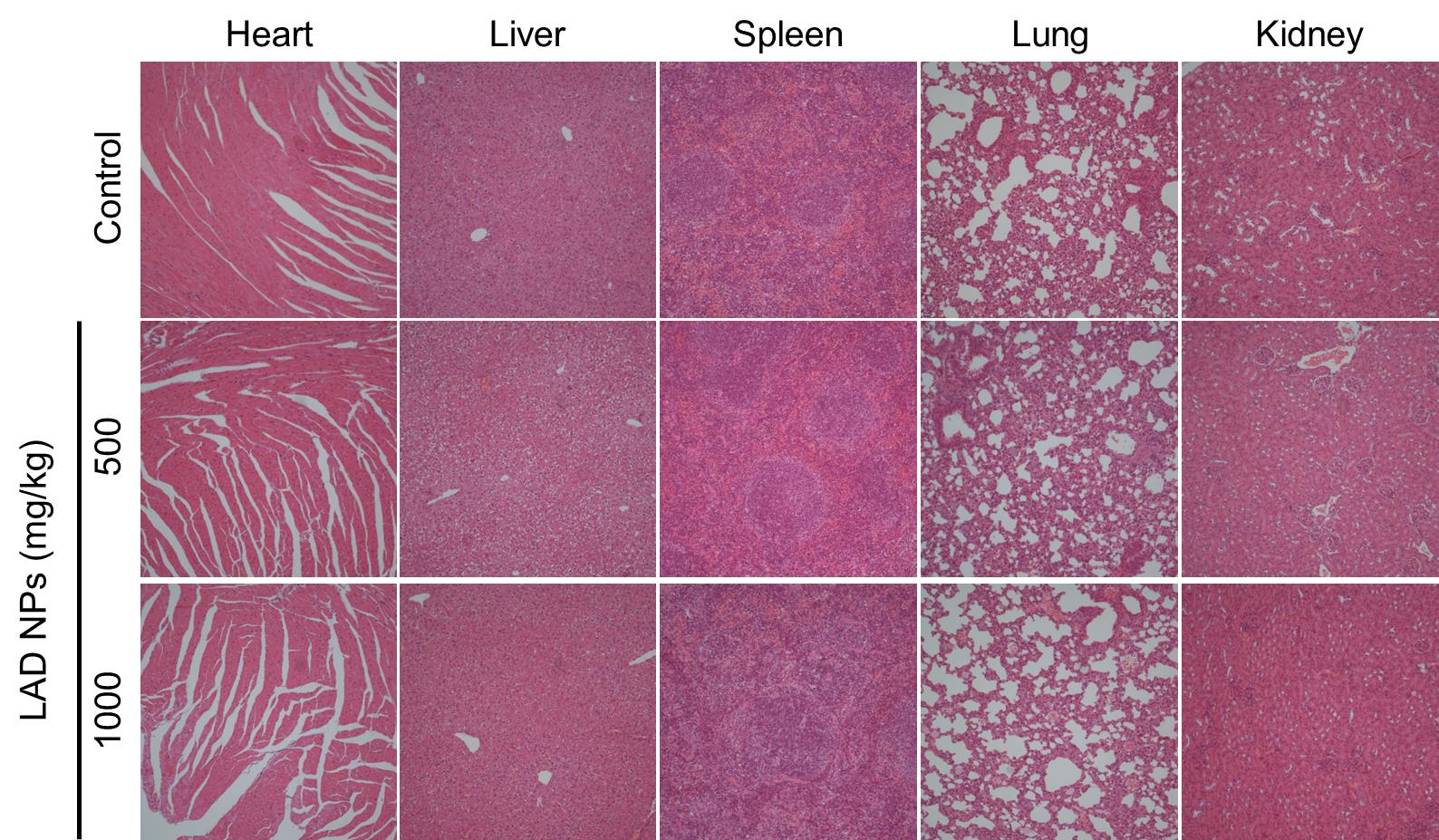


**Fig.** **S25** H&E-stained pathological sections of typical major organs.
